# T antigen–specific CD8^+^ T cells associate with PD-1 blockade response in virus-positive Merkel cell carcinoma

**DOI:** 10.1172/JCI177082

**Published:** 2024-01-30

**Authors:** Ulla Kring Hansen, Candice D. Church, Ana Micaela Carnaz Simões, Marcus Svensson Frej, Amalie Kai Bentzen, Siri A. Tvingsholm, Jürgen C. Becker, Steven P. Fling, Nirasha Ramchurren, Suzanne L. Topalian, Paul T. Nghiem, Sine Reker Hadrup

**Affiliations:** 1 Section of Experimental and Translational Immunology, Department of Health Technology, Technical University of Denmark, Kongens Lyngby, Denmark.; 2 PokeAcell Aps, BioInnovation Institute, Copenhagen, Denmark.; 3 Department of Dermatology, Department of Medicine, University of Washington, Seattle, Washington, USA.; 4 Department of Translational Skin Cancer Research, University Hospital Essen and German Cancer Consortium (DKTK), Essen, Germany.; 5 German Cancer Research Center (DKFZ), Heidelberg, Germany.; 6 Department of Dermatology, University Hospital Essen, Essen, Germany.; 7 Fred Hutchinson Cancer Center, Seattle, Washington, USA.; 8 Department of Surgery, Johns Hopkins University School of Medicine, Baltimore, Maryland, USA.; 9 Bloomberg-Kimmel Institute for Cancer Immunotherapy, Sidney Kimmel Comprehensive Cancer Center, Baltimore, Maryland, USA.

**Keywords:** Immunology, Oncology, Cancer immunotherapy, Skin cancer, T cells

## Abstract

Merkel cell carcinoma (MCC) is a highly immunogenic skin cancer primarily induced by Merkel cell polyomavirus, which is driven by the expression of the oncogenic T antigens (T-Ags). Blockade of the programmed cell death protein-1 (PD-1) pathway has shown remarkable response rates, but evidence for therapy-associated T-Ag–specific immune response and therapeutic strategies for the nonresponding fraction are both limited. We tracked T-Ag–reactive CD8^+^ T cells in peripheral blood of 26 MCC patients under anti-PD1 therapy, using DNA-barcoded pMHC multimers, displaying all peptides from the predicted HLA ligandome of the oncoproteins, covering 33 class I haplotypes. We observed a broad T cell recognition of T-Ags, including identification of 20 T-Ag–derived epitopes we believe to be novel. Broadening of the T-Ag recognition profile and increased T cell frequencies during therapy were strongly associated with clinical response and prolonged progression-free survival. T-Ag–specific T cells could be further boosted and expanded directly from peripheral blood using artificial antigen-presenting scaffolds, even in patients with no detectable T-Ag–specific T cells. These T cells provided strong tumor-rejection capacity while retaining a favorable phenotype for adoptive cell transfer. These findings demonstrate that T-Ag–specific T cells are associated with the clinical outcome to PD-1 blockade and that Ag-presenting scaffolds can be used to boost such responses.

## Abstract 2

Induction of T antigen-specific T cells in breadth and magnitude is associated with response to PD-1 blockade and can be enhanced by antigen-presenting scaffolds.

## Graphical abstract

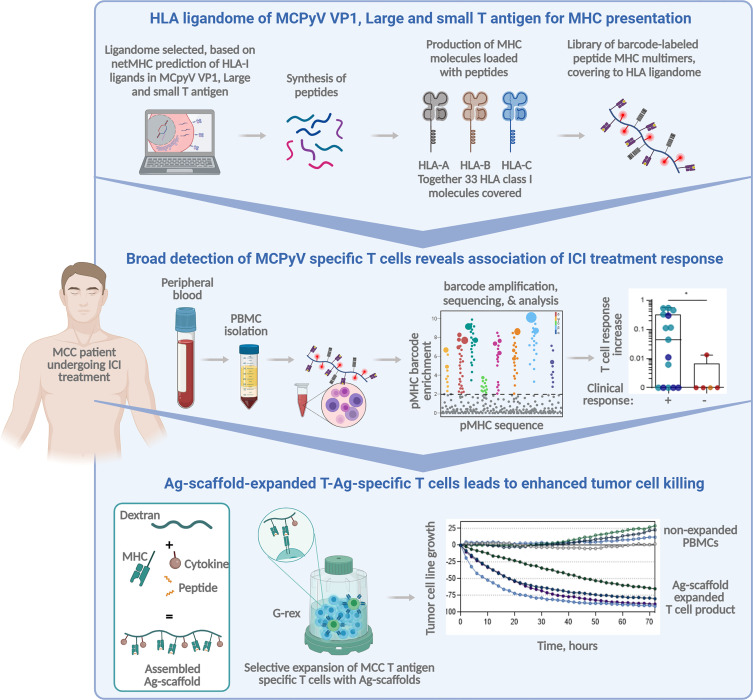

## Introduction

Immunotherapy based on blockade of the programmed cell death protein-1/programmed cell death ligand 1 (PD-1/PD-L1) pathway has demonstrated pronounced efficacy in restoring antitumor activity in some patients with solid tumors (1). The rare skin cancer Merkel cell carcinoma (MCC) has demonstrated profound responsiveness to PD-1/PD-L1 blockade, with some of the highest response rates (56%–62%) obtained among all solid cancers (2–4). Of MCC cases, 80% are caused by Merkel cell polyomavirus (MCPyV), the oncogenic potential of which requires integration into the host genome and truncation of the large T antigen (LTA), leading to the expression of the viral oncogenic T antigens (T-Ags; LTA and small T-Ag [STA]) (5). Virus-positive MCC has a low tumor mutation burden (TMB) (6), which for other tumor types is associated with lower response rates to immune checkpoint inhibition (ICI) (1). Instead, it is hypothesized that the viral T-Ags are targets for the immune recognition responsible for the high ICI response rate in this tumor type. A persistent expression of these T-Ags is essential for tumorigenesis and maintenance (7, 8), making them ideal targets for adaptive immune control. In fact, immune surveillance has already proven critical for tumor control, through positive associations between survival and intratumoral levels of both CD3^+^ and CD8^+^ lymphocytes (9, 10) and CD8^+^ T cells reactive toward KLLEIAPNC, an epitope embedded in the overlapping sequence of LTA and STA (common T-Ag [CT]) (11). Serum levels of anti–T-Ag antibodies have additionally served as an indicator of disease burden (12), and T-Ag–reactive CD8^+^ T cells are exclusively detected in MCC patients with virus-positive tumors compared with healthy donors and MCC patients with virus-negative tumors (13, 14). Furthermore, clinical evidence suggests that strong adaptive immune recognition is important for tumor control. This includes the rare events of complete spontaneous regression observed (15) together with increased incidence rates in individuals suffering from systemic immune suppression, such as those with HIV and solid organ transplant recipients (16, 17). Thus, we hypothesize that MCPyV-reactive CD8^+^ T cells play a role in the antitumor response generated by ICI in MCC patients.

Understanding the immune response to MCC during ICI therapy is important for improving therapeutic efficacy further. Across solid tumors, others have suggested that blockade of the PD-1/PD-L1 pathway leads not only to reinvigoration of preexisting dysfunctional T cells in the tumor microenvironment, but also infiltration of new tumor-reactive T cell clones (18, 19). This is supported by evidence of rapid and robust T cell proliferation in the periphery following ICI therapy (20–22). Such peripheral immune induction would be easily measurable with a minimally invasive blood-based source, which would allow fast evaluation and prediction of therapy response. In the current study, we utilized high-throughput screening technology with DNA barcode-labeled multimers (23). This allowed us to study a comprehensive panel of potential T-Ag–derived T cell epitopes, covering the full HLA ligandome and restricted to a broad range of HLA haplotypes, thereby capturing the majority of T-Ag–reactive CD8^+^ T cells present in the circulation of ICI-treated MCC patients to evaluate their response to therapy.

Despite the high response rate to PD-1/PD-L1 blockade, in the end, around half of MCC patients do not derive durable benefit from therapy, and no strong alternative therapeutic strategy for this cohort currently exists. Given the tumor-exclusive and required expression of the T-Ags, these are ideal targets for a precision-targeted T cell therapy approach. Furthermore, the T-Ags are shared across all patients and would not require personalized antigen (Ag) selection. To date, adoptive cell therapy (ACT) of single epitope expanded T cells has been clinically tested in MCC, but with limited efficacy due to acquired immune escape by HLA class I allelic loss (24, 25). Currently, high-affinity TCR-transgenic T cells targeting the CT-derived epitope, KLLEIAPNC (ClinicalTrials.gov NCT03747484) are being clinically evaluated. Thus, to leverage the knowledge of a broad repertoire of CD8^+^ T cell epitopes derived from T-Ag presented herein and in literature (13, 14, 26), we examined the capacity to expand such T-Ag–specific T cells from the peripheral blood through coordinated peptide-HLA and cytokine stimulation by artificial Ag-presenting scaffolds (27). This approach maintains a broad T cell recognition and clonality profile, while generating T cell products with advantageous phenotype and potent tumor-rejection capacity. Such a therapeutic strategy could be used to further boost the T cell compartment in patients with partial response to ICI or facilitate antitumor responses in patients with primary ICI resistance.

## Results

### Broad recognition of MCPyV-derived epitopes across a wide range of HLA haplotypes.

**Figure 1:**
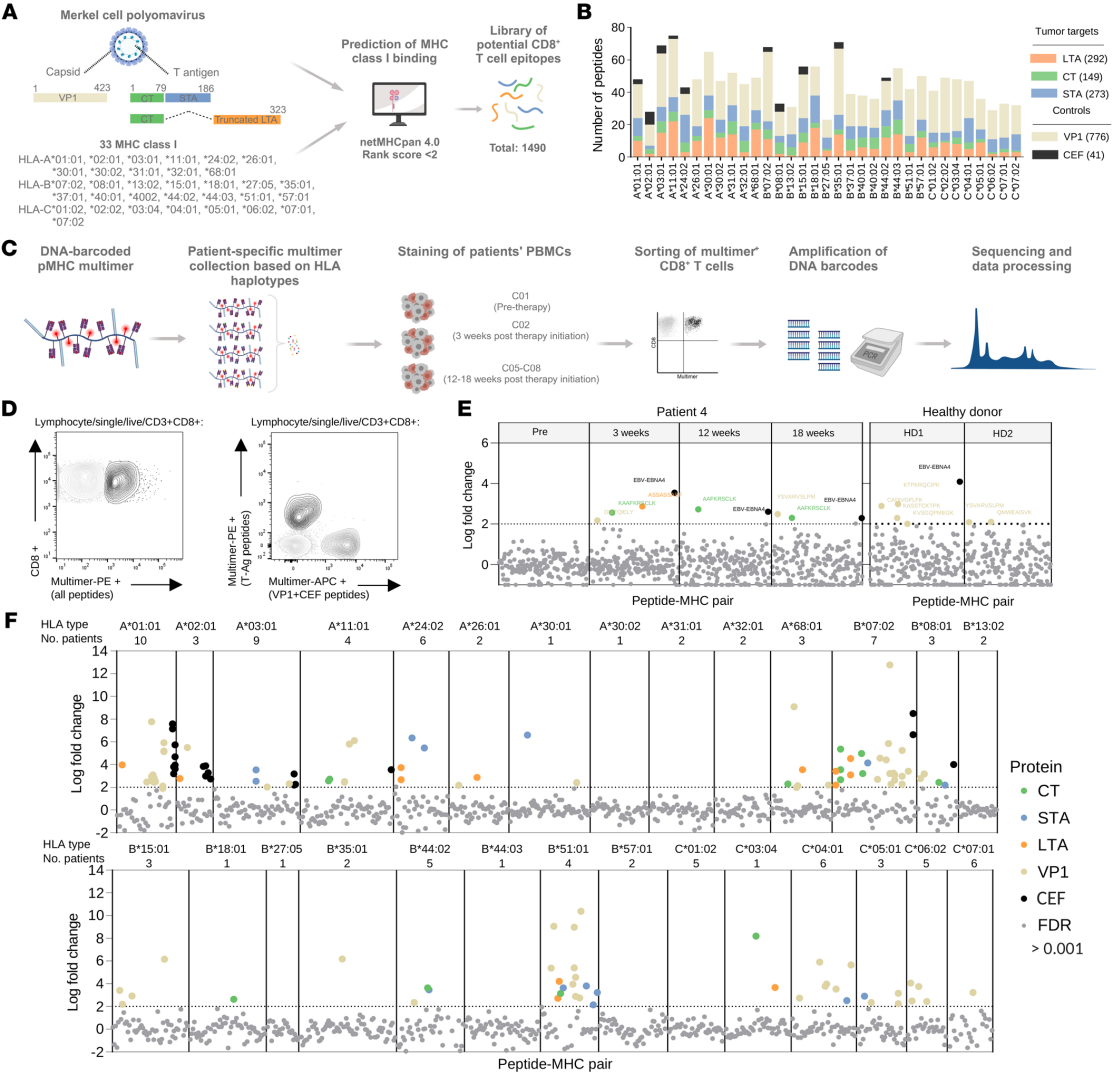
Screening with DNA barcode–labeled pMHC multimers. (**A**) Schematic overview of the in silico peptide prediction for selecting the library of MCPyV-derived peptides. Created with BioRender. (**B**) The distribution of the peptides across the 33 HLA haplotypes, colored based on the protein of origin. (**C**) Experimental workflow for the detection of multimer-reactive CD8^+^ T cells. Created with BioRender. (**D**) Flow plots for the 2 multimer design strategies with either single multimer color for all peptides (PE) (left) or 2 multimer colors separating T-Ag peptides (PE) and VP1+CEF peptides (APC) (right). (**E**) Representative examples of screening results for patient 4 and 2 non–ICI-treated healthy donors screened in parallel. T cell recognition of a given epitope is defined by significant enrichment of the pMHC-assigned DNA barcode with log-fold change > 2 and FDR < 0.001, indicated by the dotted line. T cell epitopes are colored based on the protein of origin. (**F**) Combined screening results for all patients divided based on HLA haplotype. Number of patients screened with a given haplotype is indicated above the graph.

To perform a comprehensive evaluation of circulating MCPyV-specific CD8^+^ T cells in MCC patients undergoing ICI, we first generated an extensive library of potential CD8^+^ T cell epitopes from the T-Ag proteins (truncated LTA and STA) and viral capsid protein 1 (VP1). We included the full predicted ligandome for 33 HLA class I haplotypes to ensure broad patient coverage through in silico binding prediction of 9- and 10-mer peptides to the 33 HLA class I haplotypes using netMHCpan 4.0 (28) with a predicted eluted ligand percentile rank score cutoff of 2. This resulted in 1,490 unique peptide-MHC (pMHC) complexes used for evaluation of T cell recognition, of which 714 presented T-Ag–derived peptides, and these were distributed with 7–38 peptides presented per HLA haplotype (Figure 1, A and B, and Supplemental Table 1; supplemental material available online with this article; https://doi.org/10.1172/JCI177082DS1). Additional control epitopes from common nononcogenic viruses, including CMV, EBV, and influenza (FLU), were available for 10 of the HLA haplotypes and served as technical validation for the T cell detection process (Figure 1B). These will be referred to as CEF peptides.

Peripheral blood samples from 26 patients enrolled in the Cancer Immunotherapy Trials Network CITN-09 clinical trial (ClinicalTrials.gov NCT02267603) were included with 1–4 PBMC samples obtained before and/or on anti-PD1 therapy (Supplemental Table 2). Patient blood samples were screened with HLA-matched DNA-barcoded pMHC multimers carrying the above-selected peptide library as schematically depicted in Figure 1C. Utilizing this screening technology allowed us to identify T cell recognition against a large number of pMHC specificities simultaneously while maintaining the pMHC-specificity knowledge, since every peptide specificity is identifiable by its DNA barcode tag (23). The patients’ samples were screened with 45–302 pMHC multimers covering on average 74% of their HLA class I haplotypes. The HLA haplotype C*07:02 was later excluded due to technical concerns, and 4 haplotypes (B*37:01, B*40:01/02, and C*02:02) were not represented in our patient material (Supplemental Figure 1A). For half of the patients, all multimers had a common phycoerythrin (PE) label for sorting multimer-binding CD8^+^ T cells. For the other half, 2 fluorescent labels, PE and allophycocyanin (APC), were associated with either T-Ag or the controls VP1 and CEF, respectively (Figure 1D, full gating strategy in Supplemental Figure 1B). This allowed us to include a 12-parameter T cell phenotype panel during the staining step in order to compare the phenotypes of T cells recognizing oncogenic versus nononcogenic viral elements. The associated DNA barcodes from the sorted multimer-binding cells, irrespective of their fluorescence label, were amplified and sequenced to reveal DNA barcodes enriched in the sorted T cell fraction compared with baseline levels with a false discovery rate (FDR) of less than 0.001, defining significant T cell recognition of the corresponding peptide.

The enrichment of pMHC-binding T cells (log fold change) of all pMHC multimers for patient 4 is shown in Figure 1E, with the dotted line representing the threshold of significantly enriched pMHC binding from T cells and the vertical lines separating the 4 blood samples screened. No T-Ag–specific cells were detected prior to therapy, but following ICI treatment, recognition of a CT- and LTA-derived epitope was detected, in particular the A*01:01-restricted epitope, AAFKRSCLK, which was recognized by T cells in PBMCs at all time points after treatment initiation. Additional VP1 and CEF epitopes were recognized by T cells throughout and are the only epitope types recognized in the 2 healthy donors screened in parallel as technical controls for the screening process (Figure 1E).

In total, 172 multimer-reactive CD8^+^ T cell populations were detected across all samples and protein types with restriction to 20 of the 28 included HLA haplotypes (Figure 1F). Large variations can be observed between the HLA haplotypes in terms of recognized epitopes, which may potentially be affected by the low representation of patients with certain HLA types.

### T cell reactivity detected to 32 T-Ag–derived epitopes exclusively in MCC patients.

**Figure 2:**
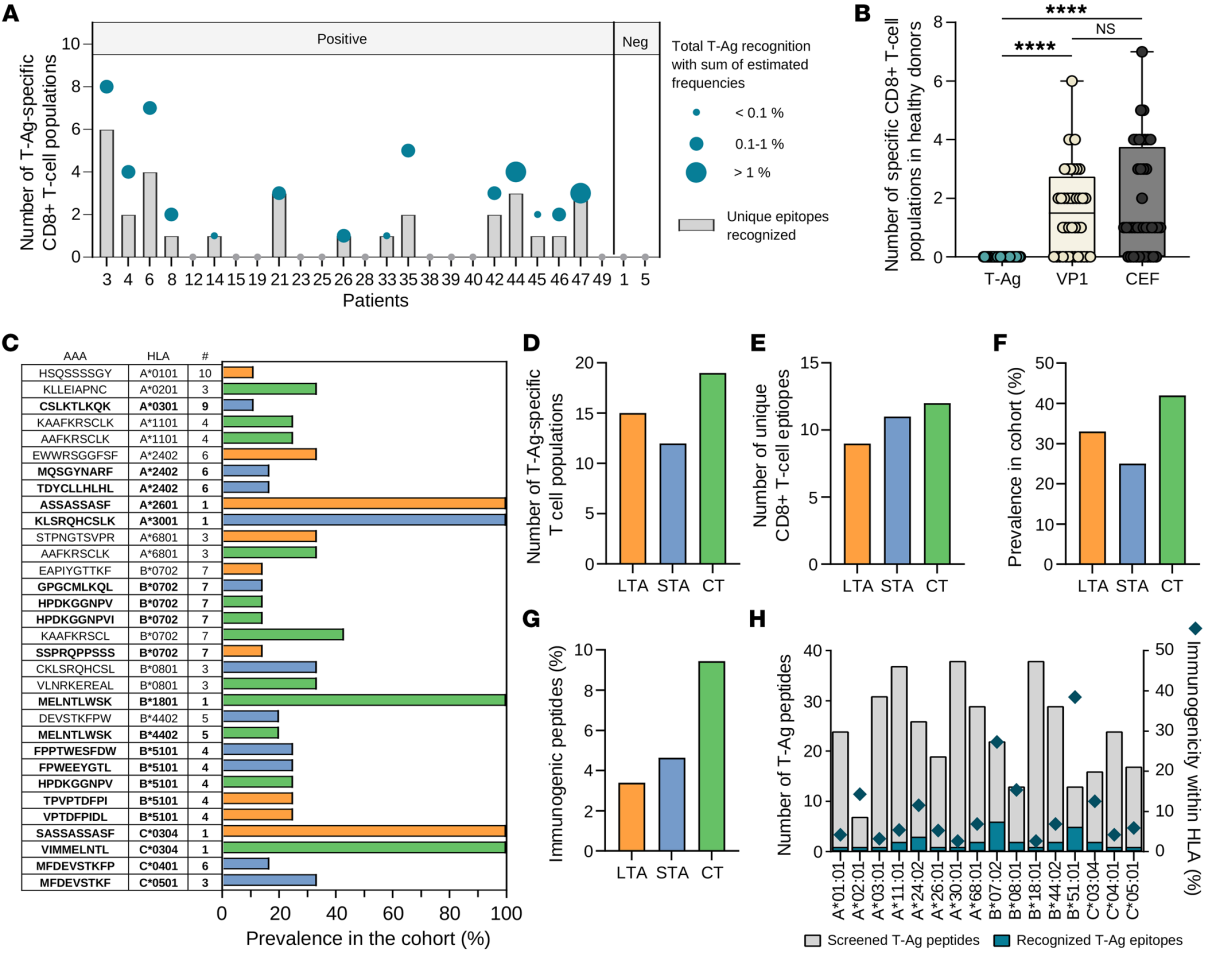
Characterization of the 32 recognized T-Ag epitopes. (**A**) The numbers of unique (bar) and total (dot) T-Ag epitopes recognized by T cells across all time points in 14 out of 26 MCC patients with tumor MCPyV status are indicated. The size of the circles varies with the summed frequency of T-Ag–specific T cells, across all time points. (**B**) T cell recognition of the 3 proteins within the healthy donor cohort (*n* = 40). ****P < 0.0001, Kruskal-Wallis test with Dunn’s correction. (**C**) Prevalence of the 32 recognized T-Ag epitopes out of screened patients with aa, HLA haplotype, and number of screened patients provided. Unreported epitopes are highlighted in bold. (**D**–**G**) Epitopes divided into their proteins of origin (LTA, orange; STA, blue; CT, green) and displayed as either total T cell populations detected (**D**), unique CD8^+^ T cell epitopes (**E**), prevalence in cohort (**F**), or immunogenic peptides out of total peptides screened within each protein (**G**). (**H**) Bars show the number of peptides screened within each HLA haplotype, with the blue fraction indicating those recognized by T cells (left *y* axis) and diamonds marking the percentage of immunogenic peptides within each HLA (right *y* axis).

Of all the multimer-reactive CD8^+^ T cell populations, 46 were T-Ag–specific, and hence tumor relevant, with 1–8 populations detected in 14 patients with MCPyV-positive tumors at summed estimated frequencies among CD8^+^ T cells ranging from 0.1% to 1% (Figure 2A). Since multiple blood samples were screened from individual patients, the number of unique T-Ag epitopes recognized per patient ranged from 1 to 6. The 2 patients with MCPyV-negative tumors had no detectable T-Ag recognition, in line with the lack of tumor expression of these oncogenes. VP1 and CEF epitopes were detectable in a large proportion of the patients as well (Supplemental Figure 1, C and D). Forty healthy donors were screened in parallel with the patient cohort, and here, no T cell recognition was observed against T-Ags, only against VP1- and CEF-derived epitopes, thus validating the tumor-specific characteristics of T-Ag expression and T cell recognition (Figure 2B), in agreement with our previous observations (13, 14).

Overall, we detected T cell recognition toward 32 T-Ag epitopes with a prevalence between 11% and 100% of the screened patients for a given HLA haplotype (Figure 2C). However, for 5 epitopes, only a single patient was screened; therefore, excluding these could give a more correct prevalence of 11% to 42%. Of the detected epitopes, 20 were previously unreported (13, 14, 26). The CT region appeared more immunogenic compared with the nonoverlapping sequences of LTA and STA, both in terms of the total numbers of T cell populations detected (Figure 2D) and the number of unique T cell epitopes derived from this region (Figure 2E). In addition, a higher proportion of patients had T cells recognizing a minimum of 1 epitope derived from the CT region (Figure 2F), and the fraction of the predicted peptides recognized by T cells (i.e., defined as immunogenic peptides) was higher for CT (Figure 2G). These observations can potentially be explained by the higher copy number of this region, since it is expressed with both LTA and STA. The T-Ag epitopes were restricted to 16 different HLA haplotypes, with B*07:02 and B*51:01 showing the highest percentages of immunogenic epitopes out of total screened peptides, (27% and 38%, respectively; Figure 2H). The immunogenic T-Ag epitopes could be distinguished from the nonimmunogenic peptides by an improved MHC-binding affinity (Supplemental Figure 2, A and B), but no difference was observed in their MHC-binding stability (Supplemental Figure 2, C and D).

### T-Ag–specific T cell populations are associated with clinical response to ICI.

**Figure 3:**
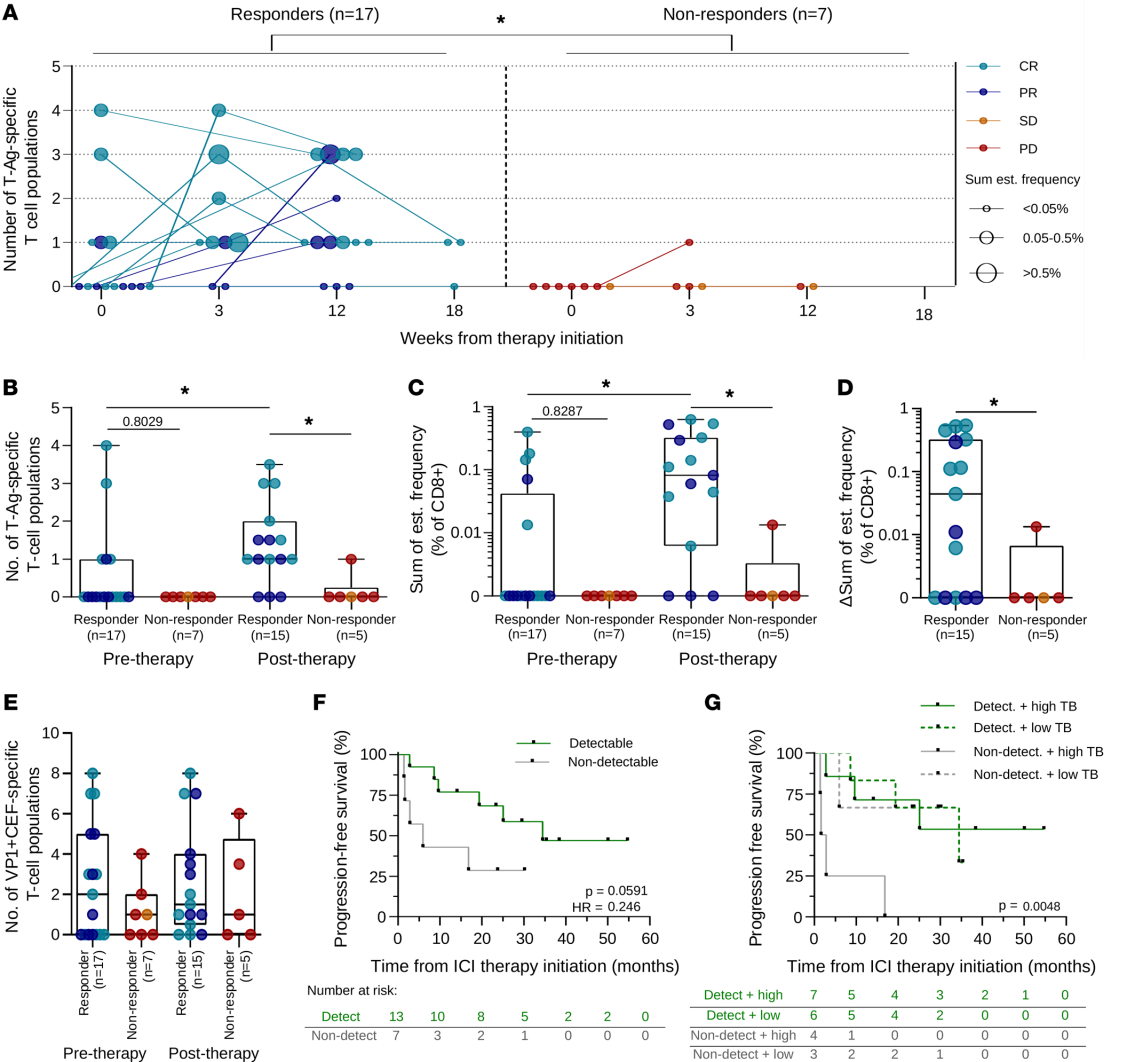
T-Ag–reactive T cells are associated with clinical benefit of ICI. (**A**) Number of T-Ag–reactive T cell populations detected during ICI therapy. The patients are divided based on their RECIST criteria into responders (CR and PR, *n* = 17) and nonresponders (SD and PD, *n* = 7) and colored accordingly with size of circles indicating the summed estimated (est.) frequency of T-Ag–reactive T cells out of CD8^+^. *P < 0.05, 2-way ANOVA. (**B**) Number of T-Ag–specific T cells detected for the 2 patient groups before and after therapy initiation. The pooled posttherapy number was based on either 3-week or 12-week time points or an average of both. *P < 0.05, Kruskal-Wallis test with Dunn’s correction. (**C**) The sum of estimated frequency of T-Ag–reactive T cells before and after therapy for patient groups. *P < 0.05, Kruskal-Wallis test with Dunn’s correction. (**D**) Change in the sum of estimated frequency before and after therapy. *P < 0.05, Mann-Whitney *U* test. (**E**) Number of VP1- and CEF-specific T cells detected for the 2 patient groups before and after therapy initiation. **B**–**E** are presented with box plots displaying the interquartile range. (**F**) Progression-free survival curves split based on detectable (*n* = 13) or nondetectable (*n* = 7) T-Ag–reactive T cells at any time point after ICI therapy initiation. Significance levels and hazard ratios are denoted; log-rank (Mantel-Cox) test. (**G**) Progression-free survival curves for detectable (Detect.) T-Ag–reactive T cells split by median baseline tumor burden (diameter = 42 mm) and for nondetectable (Nondetect.) T-Ag–reactive T cells split by median baseline tumor burden (diameter = 15 mm). Significance levels are denoted, log-rank (Mantel-Cox) test. TB, tumor burden.

To test the hypothesis that T-Ag–specific T cells contribute to tumor recognition and elimination following ICI treatment, we evaluated the association of T-Ag–restricted T cell recognition with clinical outcomes. The patients were grouped according to RECIST criteria (29), as either responders (complete response [CR] or partial response [PR]) or nonresponders (stable disease [SD] or progressive disease [PD]). The overall kinetics of the T-Ag–specific T cells during ICI therapy was different between the 2 patient groups, with a substantial increase in T-Ag–specific T cells observed only in the clinical response group (Figure 3A). Since the posttherapy blood samples were not available at all potential time points (3, 12, and 18 weeks) from several patients, the following analyses were performed with a pooled posttherapy measurement, either the 3-week sample, the 12-week sample, or an average of these 2 time points when both were available. Data from the individual time points are plotted in Supplemental Figure 3. We detected a significantly higher number of T-Ag–specific T cell populations in the clinical responders after treatment initiation (Figure 3B) compared with nonresponders. These T cell populations were enriched both in terms of their breadth of response, i.e., the number of different T-Ag–derived epitopes recognized (Figure 3B and Supplemental Figure 3A), and the magnitude of such T cell populations in the peripheral blood, i.e., the sum of estimated frequencies of T-Ag–specific T cells out of CD8^+^ T cells at each given time point (Figure 3C and Supplemental Figure 3B). Interestingly, the induction of T-Ag–specific T cells was observed early during ICI therapy for patients with a CR outcome (3–12 weeks), but seemed slightly delayed for the patients with a PR (12 weeks, Supplemental Figure 3, C and D), although it is plausible that this trend in PR patients may be related to limited patient sample availability for the 3-week time point.

In the pretreatment blood samples, we found that T-Ag was exclusively recognized in patients who later responded to treatment, yet in the majority of responding patients, such T cell populations were undetectable prior to treatment initiation. Thus, while few patients had detectable T-Ag–specific T cell populations before ICI treatment, the capacity to mount or enhance the T cell response in association with ICI therapy was significantly stronger in the responder group, as measured by the increase in T-Ag–specific T cell frequencies from before to after therapy (Figure 3D). In contrast, no differences were observed in T cell recognition of the control peptides, VP1 and CEF, that were screened in parallel (Figure 3E).

To further investigate the importance of T-Ag–reactive T cells in mediating ICI response, we evaluated progression-free survival end points. The patients were divided according to the presence or absence of detectable T-Ag–specific T cell populations after therapy initiation (Figure 3F), and each of these groups was further split based on tumor burden (Figure 3G). Those having detectable T-Ag–specific T cells showed a trend toward enhanced progression-free survival, with significant differences when including tumor burden. However, given the small-size cohort, additional adjustments for confounding factors were not feasible and may influence this trend.

### T-Ag–specific CD8^+^ T cells are marked by distinct CD39 and Ki67 expression.

**Figure 4:**
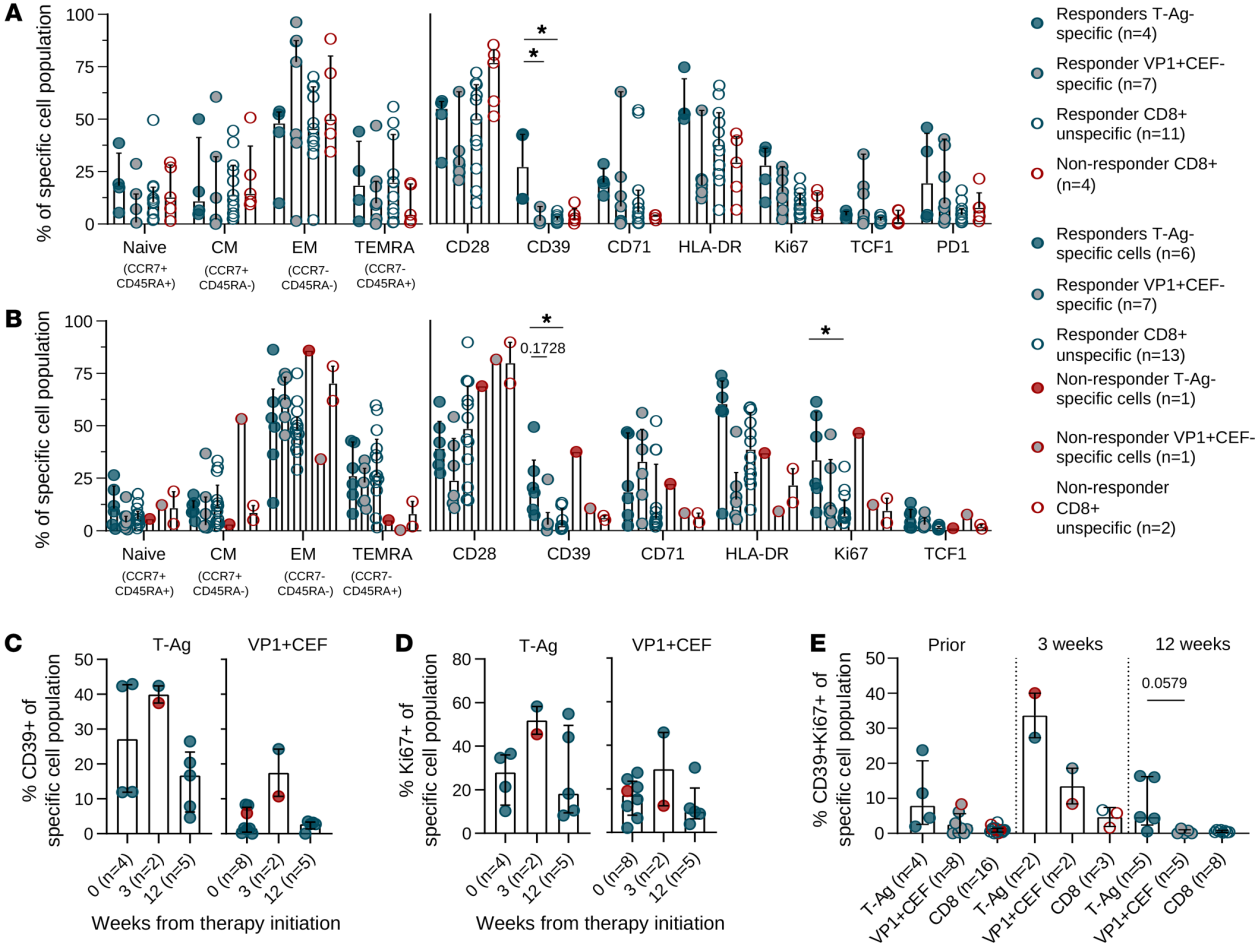
Individual marker expression before and after ICI therapy initiation. (**A** and **B**) Expression of phenotypic markers before (**A**) and after ICI therapy initiation (**B**) for responders (blue) CD8^+^ T cells with T-Ag recognition (blue filled), with VP1+CEF recognition (gray filled) or unspecific bulk cells (open circle, blue outline); and nonresponders (red) CD8^+^ T cells with T-Ag–recognition (red filled), VP1+CEF recognition (gray filled), or unspecific bulk cells (open circle, red outline). *P < 0.05, Kruskal-Wallis test with Dunn’s correction. CM, central memory. (**C**) CD39 expression on T-Ag– or VP1+CEF-specific cells divided into the 3 time points tested: prior to ICI (0 weeks) and 3 weeks and 12 weeks after ICI initiation. Kruskal-Wallis test with Dunn’s correction. (**D**) Ki67 expression on T-Ag– or VP1+CEF-specific cells divided into the 3 time points tested. Kruskal-Wallis test with Dunn’s correction. (**E**) Percentage of double-positive for CD39 and Ki67 of T-Ag–specific, VP1+CEF-specific, or unspecific CD8^+^ T cells divided into the 3 time points tested. Kruskal-Wallis test with Dunn’s correction. All bars display the median and upper quartile. Ctrl, control.

For 14 of the patients’ samples, a 12-parameter flow cytometric antibody-staining panel was employed to study T cell phenotypes associated with T-Ag–specific T cells compared with VP1- and CEF-specific T cells or the remaining bulk CD8^+^ T cells both prior to and during ICI. For the nonresponder group, no T-Ag–reactive cells were detected prior to therapy, and therefore the phenotype is provided only for bulk CD8^+^ T cells with undetermined specificities. In the responders prior to ICI therapy, T-Ag–specific T cells demonstrated significantly increased levels of CD39 (Figure 4A), indicating Ag recognition and possible exhaustion. This population also displayed a high level of HLA-DR, indicating Ag-mediated activation. The remaining phenotype characteristics were similar to the VP1- and CEF-specific and bulk CD8^+^ T cell compartments (Figure 4A). In on-treatment specimens, CD39 expression remained increased on T-Ag–specific cells compared with VP1- and CEF-specific and bulk CD8^+^ T cells, both in the responding and nonresponding patients (Figure 4B), demonstrating CD39 as a signature of T-Ag recognition in MCC patients. Interestingly, an increased expression of the proliferation marker Ki67 was observed after therapy initiation, with a significant increase in Ki67^+^ T-Ag–specific T cells compared with bulk CD8^+^ T cells following ICI therapy (Figure 4B). Dividing the samples into individual time points, irrespective of response to ICI, we found that both CD39 and Ki67 expression peaked 3 weeks after treatment initiation, followed by a decline (Figure 4, C and D). The coexpression of CD39 and Ki67 appeared specifically associated with T-Ag–specific T cells at all time points, with borderline significance at week 12 (Figure 4E).

### T-Ag–specific T cells can be expanded using artificial Ag-presenting scaffolds.

**Figure 5:**
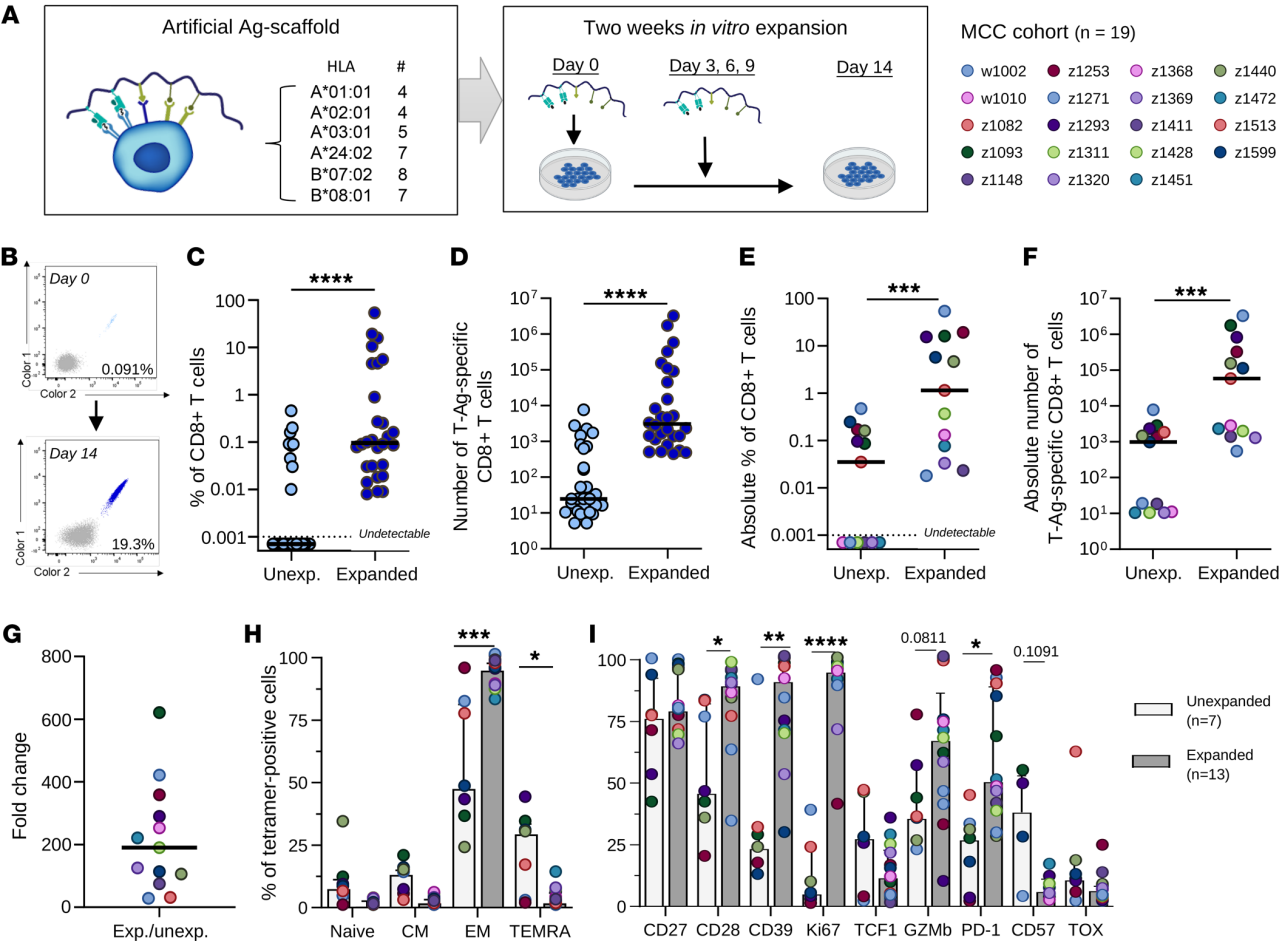
In vitro expansion of T-Ag–specific T cells using Ag-scaffolds. (**A**) Schematic illustration of the artificial Ag-scaffolds consisting of a dextran backbone with pMHC-I of interest (HLA haplotype and number of peptides presented), IL-2, and IL-21 attached, which allowed coordinated pMHC-driven stimulation (left box). During the 2 weeks in vitro expansion, Ag-scaffolds and fresh media were added to the cells on days 0, 3, 6, and 9, before the cells were harvested and evaluated on day 14 (right box). Created with BioRender. (**B**) Representative dot plots showing the expansion of RSG(A*03:01)-specific T cells. (**C** and **D**) Change in frequency (**C**) and number (**D**) of T-Ag–specific T cells (*n* = 28) during in vitro expansion. Nondetectable populations on day 0 were set to the detection limit of 0.001% of CD8^+^ T cells. ****P < 0.0001, Wilcoxon’s rank-sum test. Median displayed. (**E** and **F**) Absolute frequency (**E**) and number (**F**) of T-Ag–specific T cells in each patient (*n* = 13) before and after expansion. ***P < 0.001, Wilcoxon’s rank-sum test. Median displayed. (**G**) Fold change in the absolute number of T-Ag–specific T cells with an average of 214. (**H**) Cell-type composition of tetramer-positive cells before (light gray) and after expansion (dark gray). *P < 0.05; ***P < 0.001, Mann-Whitney *U* test. (**I**) Phenotypic marker expression on unexpanded (*n* = 7) versus expanded (*n* = 13) tetramer-positive cells. *P < 0.05; **P < 0.01; ****P < 0.0001, Mann-Whitney *U* test. Bars display the median and upper quartile. Unexp., unexpanded.

Given that T-Ags are strong tumor targets associated with MCC clearance after ICI, they may serve as ideal targets for adoptive T cell therapies. We therefore explored a recently described strategy to expand multiple T-Ag–specific T cell populations from peripheral blood using artificial Ag-presenting scaffolds (27). The Ag-scaffolds consisted of a dextran backbone carrying the pMHC of interest to allow pMHC-directed binding to specific CD8^+^ T cells as well as IL-2 and IL-21 to allow cytokine-mediated stimulation exclusively to pMHC-binding T cells in the PBMC pool (Figure 5A). The approach allowed multiple different epitope-specific T cell populations to be expanded simultaneously. We therefore selected 6 prevalent HLA-I haplotypes, A*01:01, A*02:01, A*03:01, A*24:02, B*07:02, and B*08:01, loaded with 4 to 8 T-Ag–derived epitopes each (Figure 5A). The selected epitopes were either detected in the above screening study or previously described (13, 14, 26). The in vitro cell expansion was a 2-week process with Ag-scaffolds supplemented to the cell culture on days 0, 3, 6, and 9 before the T cells were harvested and evaluated on day 14.

To study T cell expansion, a second cohort of 19 MCC patients was included with a minimum of 1 HLA-I matching the selected haplotypes and with PBMC samples available either before ICI therapy or 21–190 days after treatment initiation (Supplemental Table 3). PBMCs were expanded with 4–23 T-Ag–derived epitopes presented by the Ag-scaffolds. T-Ag–specific T cell populations could be successfully expanded in 13 of the 19 patients’ samples, resulting in a substantial increase in T-Ag–responsive T cells following the 2-week culture (Figure 5, B–D). Exemplified in Figure 5B is one such T-Ag–specific population with a frequency increase following expansion from 0.091% to 19.3% of CD8^+^ recognizing the HLA-A*0301–restricted LTA-derived epitope RSGGFSFGK. Overall, the T-Ag–specific T cell populations were significantly increased in both frequencies and absolute numbers after Ag-scaffold expansion (Figure 5, C and D). In one patient, the T-Ag–specific cells accounted for more than 50% of CD8^+^ T cells after expansion. In addition, several of the T-Ag populations were expanded from undetectable levels in the unexpanded PBMCs. To allow estimation of the number of precursor cells in these samples, the frequencies of specific cells prior to expansion were set to the detection limit of the fluorescently labeled pMHC tetramer technology (0.001% of CD8^+^ T cells; ref. 30). On a per-patient level, we observed expansion of 1 to 5 T-Ag–specific T cell populations. Thus, the sum of all T-Ag–specific T cell populations was established for each cell product, and this total pool was likewise significantly expanded in both absolute frequency and number (Figure 5, E and F) with a fold increase in the number of specific cells ranging from 29 to 621 and an average increase of 214-fold across all patients (Figure 5G). Categorizing patients by RECIST response or pre-ICI samples demonstrated expansion capacity within all groups, even nonresponders (Supplemental Figure 4B). Moreover, the patients on therapy tended to obtain similar numbers and fold changes of T-Ag–specific T cells following Ag-scaffold expansion, whereas the pre-ICI samples obtained lower of both (Supplemental Figure 4, C and D).

We evaluated the phenotypic changes in T-Ag–specific T cells associated with Ag-scaffold expansion and found that the expanded cells had an increased fraction of effector memory (EM) cells and a reduced fraction of terminally differentiated EM cells reexpressing CD45RA (TEMRA) compared with unexpanded cells (Figure 5H). The T-Ag–expanded cells showed significantly increased levels of Ag recognition (CD39, PD-1), costimulation capacity (CD28), and proliferation by Ki67 expression (Figure 5I) together with a tendency for increased cytotoxic functionality (GZMb) and decreased exhaustion by CD57. Together, these data indicated improvement in T cell functionality, suggesting applicability to adoptive T cell therapy and the potential for tumor elimination.

### Enhanced tumor cell killing by Ag-scaffold–expanded T-Ag–specific T cells.

**Figure 6:**
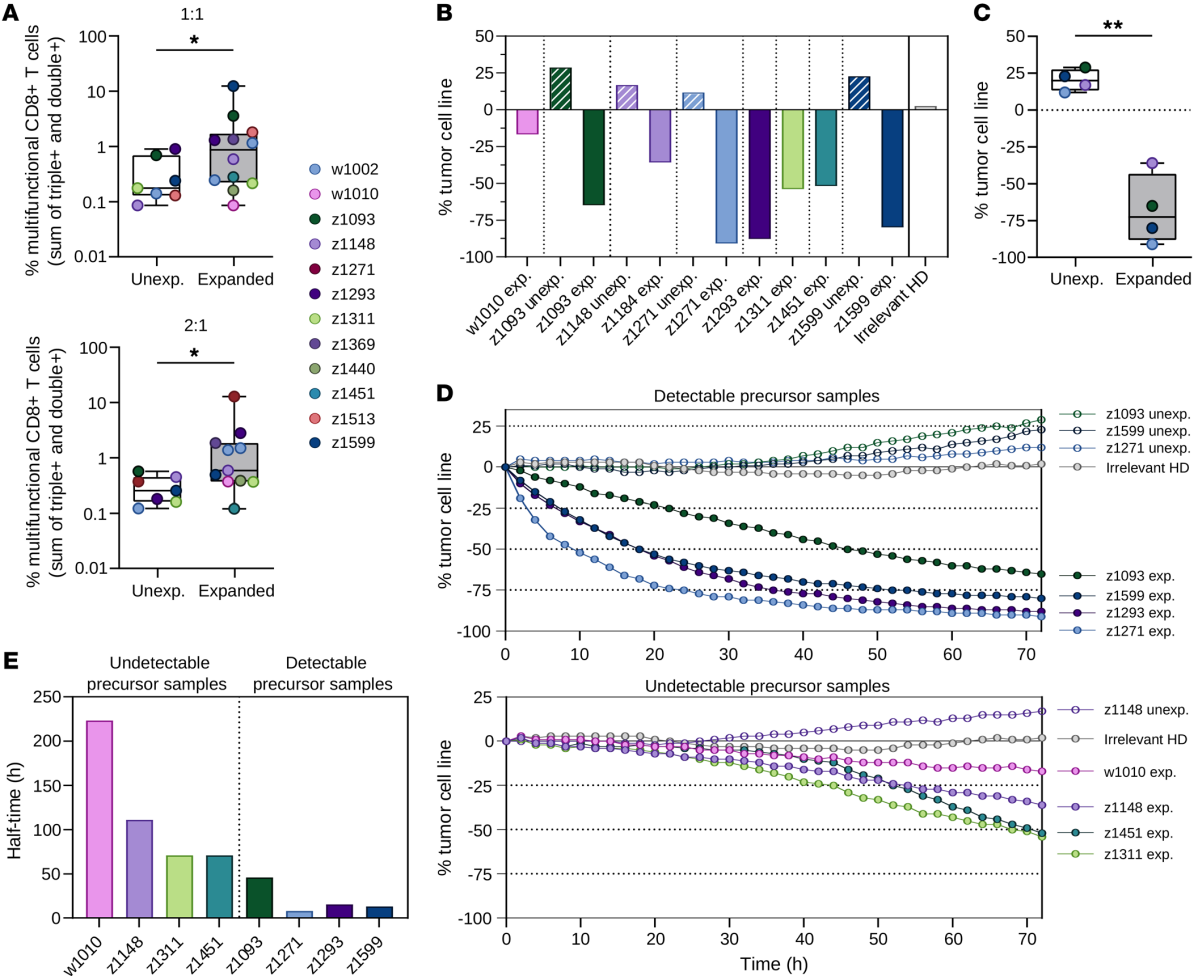
Functional capacity of T-Ag–expanded cells. (**A**) The frequencies of multifunctional CD8^+^ T cells being double- or triple-positive for IFN-γ, TNF-α, and CD107a after a 10-hour coculture between HLA-matched TCLs and either unexpanded (*n* = 7) or expanded cells (*n* = 12). The cocultures were run at 2 E:T ratios; 1:1 (top) and 2:1 (bottom). *P < 0.05, Wilcoxon’s rank-sum test. Box plots displaying the interquartile range. (**B**) The percentage of TCL change after 72-hour coculture between HLA-matched, MCC TCL, and unexpanded/expanded or irrelevant healthy donor (HD) cells run in the Incucyte instrument. (**C**) Comparison in tumor cell change between paired unexpanded and expanded cells (*n* = 4). **P < 0.01, Wilcoxon’s rank-sum test. Box plots displaying interquartile range. (**D**) Kinetics of tumor cell growth during T cell coculture for 72 hours. Patient samples have been divided based on detectable/undetectable T-Ag–specific precursor T cells prior to expansion. (**E**) Killing half-time, 50% reduction in tumor cells, for coculture with T-Ag–expanded T cell samples.

The functional capacity of the Ag-scaffold–expanded T-Ag–specific T cells in terms of tumor cell recognition and killing was assessed and compared with their unexpanded counterpart. Since no autologous tumor cells existed for these patients, the 2 allogenic MCC cell lines, WAGA and PeTa, were utilized since they matched the 6 HLA haplotypes displayed by the Ag-scaffolds and are grown in single-cell suspension. The cell lines presented no natural MHC-I expression, which could be restored by prestimulation with IFN-γ with high expression measurable for up to 72 hours (Supplemental Figure 5A). First, we conducted cocultures between expanded/unexpanded T cell pools and HLA-matched tumor cell lines (TCLs) for 10 hours, followed by measurement of intracellularly captured cytokines, IFN-γ, and TNF-α, and cytolytic degranulation through the marker CD107a in the CD8^+^ T cells. The Ag-scaffold–expanded T cells showed significantly higher frequencies of multifunctional, i.e., double- and triple-positive, CD8^+^ T cells as a response to tumor cell stimulation compared with unexpanded cells (Figure 6A). The T-Ag–specific T cells thereby retained cytotoxic functionality after Ag-scaffold expansion, which has been found to be impaired following other ex vivo expansion strategies (27).

Next, we evaluated the direct tumor cell–killing capacity by real-time monitoring of fluorescently labeled target cells upon coculture with Ag-scaffold–expanded effector cells utilizing the Incucyte instrument. The MCC cell lines, WAGA and PeTa, were transduced with a lentivirus construct encoding GFP to create stable GFP-expressing cell lines (Supplemental Figure 5B). Expanded T cells available from 8 patients were included, and for 4 of these, unexpanded cells were analyzed in parallel. Tumor-killing capacity was evaluated during a 72-hour coculture between expanded/unexpanded cells and HLA-matched, GFP-labeled MCC TCLs, in 3 different effector/target (E:T) ratios. The GFP integrated intensity was measured for each well during the duration of the assay (patient example in Supplemental Figure 5C) and used to determine the fraction of live tumor cells throughout the coculture as a measurement of tumor cell killing, compared with the wells containing tumor cells alone. All Ag-scaffold–expanded cells were capable of killing MCC cell lines, with 20%–95% cell line reduction, i.e., tumor cell death measured after 72 hours (Figure 6B). Tumor cell killing was strongly improved by the Ag-scaffold expansion, since the unexpanded cells did not affect tumor cell growth, whereas the cultures with expanded T-Ag–specific T cells provided tumor cell killing in all cases (Figure 6, B and C). To further evaluate the kinetics of tumor cell killing, the samples included were divided into detectable precursor samples, with T-Ag–specific CD8^+^ T cells expanded from detectable levels to above 1%, and undetectable precursor samples, where T-Ag–specific T cells had been expanded from undetectable levels to below 1% of CD8^+^ T cells. The killing curves for the 2 groups at the 11:1 E:T ratio showed that the samples with detectable precursors had a faster killing rate, achieving 65%–95% tumor cell reduction after 72 hours (Figure 6D) and with 50% killing obtained already after 8–46 hours (Figure 6E). The low T-Ag–frequency samples still obtained 20%–50% killing after 72 hours, with an estimated 50% killing between 71 and 211 hours. A similar pattern of results was observed at the lower E:T ratios despite being at a slower pace (Supplemental Figure 5, D and E). Cocultures with CMV-expanded cells as effectors were run in parallel as a control for density-dependent tumor cell killing and did not show any effect on tumor growth (Figure 6, B and C, and Supplemental Figure 5, D and E).

Overall, these assays demonstrated that T-Ag–specific T cells can be expanded from peripheral blood to significant numbers and provide substantially better tumor-killing potential than the original, unexpanded PBMC sample. Hence, Ag-scaffold–driven T-Ag–specific T cell expansion can be used to enhance CD8^+^ T cell reactivity to these critical tumor Ags and establish a T cell effector pool even from patients with no detectable T-Ag–specific T cells prior to expansion.

## Discussion

Understanding the immune response to MCC during ICI therapy is important for our understanding of immunologic events underlying blockade of the PD-1 pathway. This knowledge can be used as a predictive biomarker for patient stratification where a blood-based source would be highly favorable, since it is minimally invasive and easily accessible compared with a tumor biopsy. Moreover, expanding our knowledge related to T-Ag–derived epitopes of relevance for T cell recognition and tumor cell killing is critical for the development of new immunoprecision therapeutic strategies targeting such Ags.

In the present study, we utilized a high-throughput screening technology that allowed us to study a comprehensive panel of potential T-Ag–derived T cell epitopes restricted to 33 HLA haplotypes, which covered, on average, 74% of the patients’ HLA haplotypes. In our investigation of 714 T-Ag–displaying pMHC complexes, we detected T cell recognition of 32 unique epitopes. Among these, 20 were previously unreported (13, 14, 26), which also included epitopes presented in the context of 6 new HLA haplotypes not, to our knowledge, evaluated in MCC before. Thus, the T-Ag–derived epitope landscape has been substantially extended in both amount and HLA coverage, which can benefit future biomarker screening and potential targeted therapeutic approaches.

The breadth and magnitude of circulating T-Ag–reactive T cells were enhanced by ICI therapy specifically in clinical responders. The kinetics of the T-Ag–reactive T cells indicated a rapid increase during the first 3 weeks followed by a decrease at the later time points for complete responders, whereas partial responders showed a later increase detectable at the 12-week time point. However, only a limited number of 3-week samples were available for this patient group, and it would therefore be valuable to strengthen this finding. This kinetic of a rapid increase in peripheral tumor-specific T cells within the first 3 weeks is in line with published observations made in several other solid cancer types (21, 31–34). In one of these studies, neoepitope-specific T cells have been directly measured in the peripheral blood and correlated with response to PD-L1 blockade in patients with metastatic urothelial carcinoma (32). Still, most studies rely on phenotypic markers as a surrogate for tumor reactivity during PD-1/PD-L1 blockade, and positive associations with T cell markers, such as CD39, Ki67, PD-1, TCF1, and CD103, have been reported (21, 32–36). In our investigation, CD39 appeared as a signature of T-Ag recognition in the peripheral of the 14 patients evaluated, which has also been reported to define tumor-specific exhausted CD8^+^ T cells in tumor-infiltrating lymphocytes (TIL) (35–37). In response to PD-1 blockade, elevated levels of double-positive CD39^+^Ki67^+^–expressing cells were found to be T-Ag–specific compared with the remaining T cell compartment, thereby marking them as a distinct proliferative blood-based reservoir of antitumor effector cells. However, it was not feasible to predict whether this on-therapy phenotype was associated with clinical outcome due to the limited sample availability, especially at the 3-week time point that appeared most important for complete tumor clearance.

In the pretreatment setting, T-Ag–specific T cells were only detectable in patients who later responded, suggesting this as a specific marker for identifying patients likely to respond. However, a large proportion of patients in our cohort failed to display T cell reactivity to T-Ag prior to treatment initiation, but developed this during therapy. Additional studies are currently investigating the role of T-Ag–specific T cell responses related to ICI in MCC and have demonstrated T-Ag–specific T cells as a prognostic biomarker prior to immunotherapy (38, 39). Still, analysis of larger cohort sizes would be beneficial to validate these findings.

In the current study, we did not investigate the potential role of CD4^+^ T cells in response to ICI therapy due to technical limitations in MHC-II molecule synthesis and prediction of relevant peptides for tetramer/multimer technologies. Still, this cell subset could be highly relevant, with increasing numbers of investigations indicating its roles in tumor control, both as helpers for CD8^+^ T and B cells and as direct mediators of antitumor cytotoxicity (40). Furthermore, the CD4 compartment has been positively associated with response to ICI therapy (41–43), and a recent study in MCPyV-negative MCC even showed a positive association between neoantigen-specific CD4^+^ T cells in response to PD-1 blockade (44). To date, 7 T-Ag–derived CD4^+^ T cell epitopes have been described, but not correlated with therapy outcome (45).

The oncogenic T-Ags serve as highly tumor-specific targets in MCC that may be ideal for precision targeting with adoptive cell therapy. To date, only single-epitope–directed approaches have been explored in the clinic using ex vivo–expanded T cells (24, 25) or high-affinity TCR-transgenic T cells (trial running at this writing: ClinicalTrials.gov NCT03747484). However, such single-targeted strategies are prone to tumor escape due to high selection pressure and Ag loss variants, which was also concluded for the first trial (24, 25). We therefore assessed a multitargeted approach using artificial Ag-presenting scaffolds displaying T-Ag–derived epitopes together with critical cytokines (27). Using this strategy, multiple pMHC-specific T cell populations can be expanded simultaneously while maintaining polyclonal TCR characteristics within each population (27). We successfully expanded between 1 and 5 T-Ag–specific T cell populations in 13 out of 19 patients’ PBMCs with an average 214-fold increase in total T-Ag–specific cell numbers. The T-Ag–expanded cells displayed an improved phenotype, i.e., higher activation, cytotoxicity, and less exhaustion compared with unexpanded cells, and also demonstrated strong tumor cell killing, which was substantially enhanced compared with the original unexpanded PBMC sample. It is noteworthy that several of the T-Ag populations were expanded from undetectable levels in the unexpanded PBMCs, and even 6 of the patients had no detectable T-Ag recognition at all prior to expansion. Still, these expanded cell products could mediate tumor cell killing, which is of high relevance, as this patient group is less likely to respond to ICI therapy, and suggests that Ag-scaffold–mediated expansion of T-Ag–specific T cells could be a beneficial treatment strategy for the nonresponding patient group, potentially in combination with an HLA upregulation agent to limit this immune evasion. Several such agents have already been investigated in MCC (46, 47). Other approaches to targeting multiple tumor Ags have been explored, such as adoptive transfer of TILs, where the cell product reflects the tumor infiltrations and may respond to a broad set of Ags. This strategy is well explored and clinically validated in melanoma (48), but no reports are published related to MCC.

In summary, our study provides important insights into the dynamics of T-Ag–specific CD8^+^ T cell responses during PD-1 blockade. These Ag-reactive cells play a crucial role in tumor cell killing and are associated with a clinical outcome, as highlighted by the fact that an increased breadth and magnitude of such responses within the first weeks of treatment are observed in patients responding to therapy. Further investigation is required in order to define a prognostic biomarker to predict the clinical benefit of ICI therapy to better stratify patients accordingly. Our findings suggest a tendency for increased levels of preexisting T-Ag–specific cells in responding patients. Furthermore, we demonstrated that the technology of artificial Ag-presenting scaffolds allowed us to highly amplify and functionally improve T-Ag–reactive T cells. For such an approach, the T-Ag–derived epitopes described herein and in the literature serve as ideal tumor-specific targets. The combinations of Ag-scaffold–expanded T cell products and ICI could be relevant as a novel therapeutic strategy, particularly for patients not mounting sufficient T cell response to T-Ag with ICI therapy alone.

## Methods

### Sex as a biological variable.

Sex was not considered as a biological variable in this study, and the experiments and data analyses were conducted blinded to the sex of the studied subjects. The sex of the patients was not revealed to us.

### Patient and healthy donor material.

All MCC patient samples were provided by the Fred Hutchinson Cancer Center. Cohort 1 consisted of 26 patients receiving pembrolizumab every 3 weeks on the CITN-09/Keynote-017 trial (ClinicalTrials.gov NCT02267603). Blood samples were drawn at 1 to 4 time points during therapy (C01, prior; C02, 3 weeks after; C05, 12 weeks after; C08, 18 weeks after). An additional cohort 2 consisting of 19 patients was included for expansion with Ag-presenting scaffolds with a single blood sample drawn either early during ICI therapy (*n* = 13) or prior to any immunotherapy (*n* = 6). Clinical information with RECIST criteria obtained following ICI therapy is presented in Supplemental Table 2 (cohort 1) and Supplemental Table 3 (cohort 2). Healthy donor blood samples were obtained from the blood bank at Rigshospitalet (Copenhagen, Denmark).

PBMCs from both MCC patients and healthy donors were isolated from whole blood by Ficoll density gradient centrifugation at 1,000*g* and cryopreserved in liquid nitrogen. All samples were HLA class I, genotyped by either Bloodworks Northwest (Seattle, Washington, USA) or DKMS Life Science (Dresden, Germany).

### TCLs.

The allogeneic MCC TCLs, WAGA and PeTa, were provided by the Department of Translational Skin Cancer Research, University Hospital Essen, and mycoplasma tested upon arrival. The TCLs were HLA genotyped by DKMS Life Science, with the following HLA haplotypes (HLA-A and HLA-B only): WAGA: A*01:01, A*02:01, B*07:02, and B*08:01; PeTa: A*03:01, A*11:01, B*35:01, and B*35:02.

### MCPyV peptide selection.

The truncated LTA (323 aa) and STA (186 aa) obtained from isolate MCC348 (GenBank FJ173809.1) served as a source of potential tumor-specific T cell targets whereas VP1 (423 aa) obtained from isolate MLK-1 (GenBank FJ173815.1) was a control for MCPyV infection. MHC-I–binding peptides were predicted as 9 and 10 mer with an eluted ligand percentile rank score (EL %Rank) below 2 using NetMHCpan 4.0 (28) against 11 HLA-A, 14 HLA-B, and 8 HLA-C molecules (Supplemental Table 1). All selected peptides and CEF peptides were purchased from Pepscan (Pepscan Presto BV) and dissolved to 10 mM in DMSO.

### MHC monomer production and generation of specific pMHC complexes.

The production of biotinylated MHC-I monomers was performed as previously described (49–51) in the presence of a UV-sensitive ligand (52, 53) or as empty peptide-receptive molecules (51). Specific pMHC complexes were generated by either 1-hour UV-induced peptide exchange or direct peptide loading to the empty MHC molecules.

### Detection of pMHC-specific T cells using DNA-barcoded multimers.

MCPyV- and virus control–specific T cells were detected using DNA-barcoded pMHC multimers as previously described (23). Unique DNA barcodes were first coupled to either PE- or APC-labeled dextran backbones (Fina Biosolutions) for 30 minutes at 4°C, followed by the addition of relevant pMHC complexes for 30 minutes at 4°C, thereby assigning a unique DNA barcode and a common fluorescent label to each pMHC specificity. Patient samples and healthy donor PBMCs were stained with an upconcentrated pool of HLA-matched multimers in the presence of 50 nM dasatinib for 15 minutes at 37°C. Two different antibody stainings were performed on the samples; the first half of the cohort was stained with antibody panel I (Supplemental Table 4) and a dead cell marker (LIVE/DEAD Fixable Near-IR; Invitrogen, L10119) for 30 minutes at 4°C and fixed in 1% paraformaldehyde (PFA). The second part of the cohort was first stained with surface markers (panel II, Supplemental Table 4) and a dead cell marker (Near-IR) for 30 minutes at 4°C, then permeabilized with a Foxp3/Transcription Factor Staining Buffer Set (eBioscience, 11500597) and stained with the intranuclear markers (panel II, Supplemental Table 4) for 60 minutes at room temperature before fixation in 1% PFA. Multimer-binding T cells were sorted as lymphocytes, single, live, CD8^+^, FITC^–^, or BV786^+^, and multimer^+^ (PE^+^ or APC^+^) (full gating strategy in Supplemental Figure 1B) and pelleted by centrifugation at 5,000*g*. DNA barcodes from the isolated cells and a stored aliquot of the multimer pool (baseline) were amplified by PCR using the Taq PCR Master Mix Kit (QIAGEN, 201443). PCR products were purified with the QIAquick PCR Purification Kit (QIAGEN, 28104) and sequenced using an Ion Torrent PGM 316 or 318 chip (Life Technologies) at PrimBio Research Institute (Garnet Valley, Pennsylvania, USA). Sequencing data were processed using the software package Barracoda, version 1.0 (https://services.healthtech.dtu.dk/services/Barracoda-1.0/). Briefly, Barracoda identified and assigned sample ID and pMHC specificity to each DNA barcode and counted the total number of reads and clonally reduced reads for each. Fold changes in read counts were estimated by mapping read counts in a given sample relative to the mean read counts of the triplicate baseline samples using normalization factors determined by the trimmed mean of M-values method. A threshold of at least 1/1,000 reads associated with a given DNA barcode relative to the total number of DNA barcode reads in that given sample was set to avoid false-positive hits. FDRs were estimated using the Benjamini-Hochberg method, and pMHC-associated DNA barcodes enriched in the sorted population, with an FDR < 0.001, equal to *P* < 0.001, were considered to assign true T cell responses. An estimated cell frequency was calculated for each DNA barcode from their read count fraction out of the percentage of CD8^+^ multimer^+^ T cells. DNA barcodes enriched in all simultaneously screened samples, including patients and both HLA-matching and nonmatching healthy donor controls, were excluded as potential nonspecific binding.

### In vitro Ag-scaffold expansion.

Patients’ PBMCs were expanded in vitro using artificial Ag-scaffolds (27) presenting selected T-Ag epitopes. Ag-scaffolds were assembled by mixing streptavidin-conjugated (SA-conjugated) dextran backbone (500 kDa, FINA Biosolutions or Agilent Technologies) with pMHC of interest and cytokines (IL-2 and IL-21, Acro Biosystems, IL-2H82F3, and IL-21-H82F7) and incubated for 30 minutes at 4°C, followed by incubation with d-biotin for 20 minutes at 4°C. Ag-scaffolds were filtered through 100 kDa spin columns (Vivaspin6, Sartorius), mixed with a 10× Freeze Buffer (BSA+10% Glycerol), and stored at –80°C. Patients’ PBMCs were plated in a G-Rex 24 (Wilson Wolf, P/N 80192M) in X-VIVO 15 media (Lonza, BE02-060Q) supplemented with 5% human serum (Sigma-Aldrich, H4522) and stimulated with HLA-matched Ag-scaffolds. The cells were expanded for 14 days with media exchange and Ag-scaffold addition on days 3, 6, and 10 before the T cell specificity and phenotype were evaluated on day 14 as described below.

### Detection of pMHC-specific T cells using combinatorial encoded pMHC tetramers.

Peptide specificity and phenotype assessment of the unexpanded and expanded PBMCs was elucidated by combinatorial encoded fluorescently labeled pMHC tetramers (30). The pMHC complexes were multimerized on 2 different SA-conjugated fluorochromes (Supplemental Table 4) to assign a unique 2-color combination to each specificity for specificity staining or a pooled 2-color combination for all specificities to allow phenotype assessment. The samples were stained with a pool of HLA-matched tetramers in stain buffer (BD, 566349) and 50 nM dasatinib for 15 minutes at 37°C. For specificity staining, the cells were stained with antibody panel III (Supplemental Table 4) and a dead cell marker (Near-IR) for 30 minutes at 4°C. For phenotype assessment, an extended antibody panel IV (Supplemental Table 4) and dead cell marker (Near-IR) were used to stain the cells for 30 minutes at 4°C, after which they were permeabilized with a Foxp3/Transcription Factor Staining Buffer Set (eBioscience, 11500597) and stained against the intranuclear markers (panel IV) for 60 minutes at room temperature before analysis. Tetramer-binding T cells were gated as lymphocytes, singlets, live, CD8^+^CD3^+^, tetramer color1^+^, tetramer color2^+^, and negative for the remaining colors (Supplemental Figure 4A) and defined as a T cell response if a minimum of 10 dual-color positive events was detected.

### Functional assessment of T-Ag–expanded cells by intracellular cytokine staining.

The functional capacity of T-Ag–expanded T cells against allogeneic MCC TCLs was tested by intracellular cytokine staining (ICS). The TCLs (WAGA and PeTa) were stimulated with 25 IU/mL IFN-γ (PeproTech) for 24 hours to increase MHC expression levels. The expanded and unexpanded cells (if available) were cocultured with HLA-matched TCL at 1:1 and 2:1 E:T ratios in the presence pf 1 μl/mL GolgiPlug and CD107a-PE (BD Bioscience. 555801) for 10 hours at 37°C and 5% CO_2_. In parallel, effector cells were also incubated with or without a leukocyte activation cocktail (LAC, BD Bioscience) as a positive or negative control, respectively. Following incubation, cells were stained with extracellular surface antibodies (panel V, Supplemental Table 4) and dead cell marker (Near-IR) for 30 minutes at 4°C and left overnight in fixation buffer (eBioscience). The cells were permeabilized with 10× permeabilization buffer (eBioscience) and stained with intracellular antibodies from panel V for 30 minutes at 4°C before analysis. Activated cells were gated as lymphocytes, single, live, CD8^+^CD3^+^, and TNF-α/IFN-γ/CD107a double or triple positive.

### Direct killing assessment of T-Ag–expanded cells by Incucyte.

The MCC TCLs, WAGA and PeTa, were transduced with a GFP-expressing lentivirus construct provided by Unikum Therapeutics and sorted based on GFP signal. The cell-culture plate was first coated with poly-l-ornithine (Merck, P4957) for 1 hour at room temperature, removed, and left drying for an additional 30 to 60 minutes. The TCLs had been prestimulated with 25 IU/mL IFN-γ for 24 hours prior to the coculture and plated out at a density of 40,000 cells/well. The expanded and corresponding unexpanded cells, if available, were plated out in duplicates with the relevant HLA-matched TCL at E:T ratios of 4:1, 8:1, and 11:1. Wells with effector alone, TCL alone, and coculture with irrelevant effector cells were included as negative controls. Additional wells with TCLs alone in the presence of 1% Triton X-100 (Sigma-Aldrich, X100) served as positive killing controls. The cell-culture plate was placed in the Incucyte S3 Instrument (Sartorius) set to acquire 4 pictures per well through a ×20 objective every second hour for a total duration of 72 hours. All analyses were performed using Incucyte Live-Cell Analysis Software, version 2022B.

### Flow cytometry.

The flow cytometry experiments were carried out using either FACSMelody, FACSAria Fusion, or LSR Fortessa instruments (BD Biosciences). Data were analyzed using FlowJo, version 10.8.1 (TreeStar).

### Statistics.

The graphing and statistical analyses were conducted using GraphPad Prism, version 9.4.0. All data were assessed for a normal distribution with a D’Agostino-Pearson normality test. Nonparametric unpaired data were analyzed with either unpaired Mann-Whitney *U* test (Figure 3, C, E, and F, Figure 5, H and I, and Supplemental Figure 2, A–D) and Kruskal-Wallis test with Dunn’s correction analysis (Figure 2B, Figure 3, B and D, Figure 4, A and B, Supplemental Figure 3B, and Supplemental Figure 4, B and C) for single variable or 2-way ANOVA for multiple variables (Figure 3A). Nonparametric paired data were analyzed with Wilcoxon’s rank-sum test (Figure 5, D–F, and Figure 6, A and C). A *P* value of less than 0.05 was considered significant.

### Study approval.

All MCC patient blood samples were collected by Fred Hutchinson Cancer Center under approval of their Institutional Review Board. Cohort 1 consisted of patients on the CITN-09/Keynote-017 trial (ClinicalTrials.gov NCT02267603). All patients signed a written consent form according to the Declaration of Helsinki. Healthy donor blood samples were obtained from the blood bank at Rigshospitalet under approval of the Scientific Ethics Committee of the Capital Region, Denmark.

### Data availability.

Values for all data points in graphs are reported in the Supporting Data Values file. Additional raw data files can be obtained from upon request.

## Author contributions

UKH conceptualized the project, designed and performed experiments, analyzed data, created figures, and wrote the manuscript. CDC identified patient samples and discussed project plans and data. AMCS and MSF provided practical support and discussed data. AKB and ST provided technical guidance. JCB provided the MCC TCLs. SPF, NR, and SLT discussed project plans and data. PTN conceptualized the project, provided patient samples, and discussed data. SRH conceptualized the project, designed experiments, and supervised and wrote the manuscript. All authors revised and approved the manuscript.

## Supplementary Material

Supplemental data

Supplemental table 1

Supporting data values

## Version 1 01/30/2024

In-Press Preview

## Version 2 04/15/2024

Electronic publication

## Version 3 04/19/2024

Updated figure 5
