# Supplementary material for: T antigen–specific CD8+ T cells associate with PD-1 blockade response in virus-positive Merkel cell carcinoma
Source: J Clin Invest. 2024 Jan 30;134(8):e177082. doi: 10.1172/JCI177082 (PMC11014655; doi:10.1172/JCI177082)
Supplement: Supplemental data [file jci-134-177082-s007.pdf]

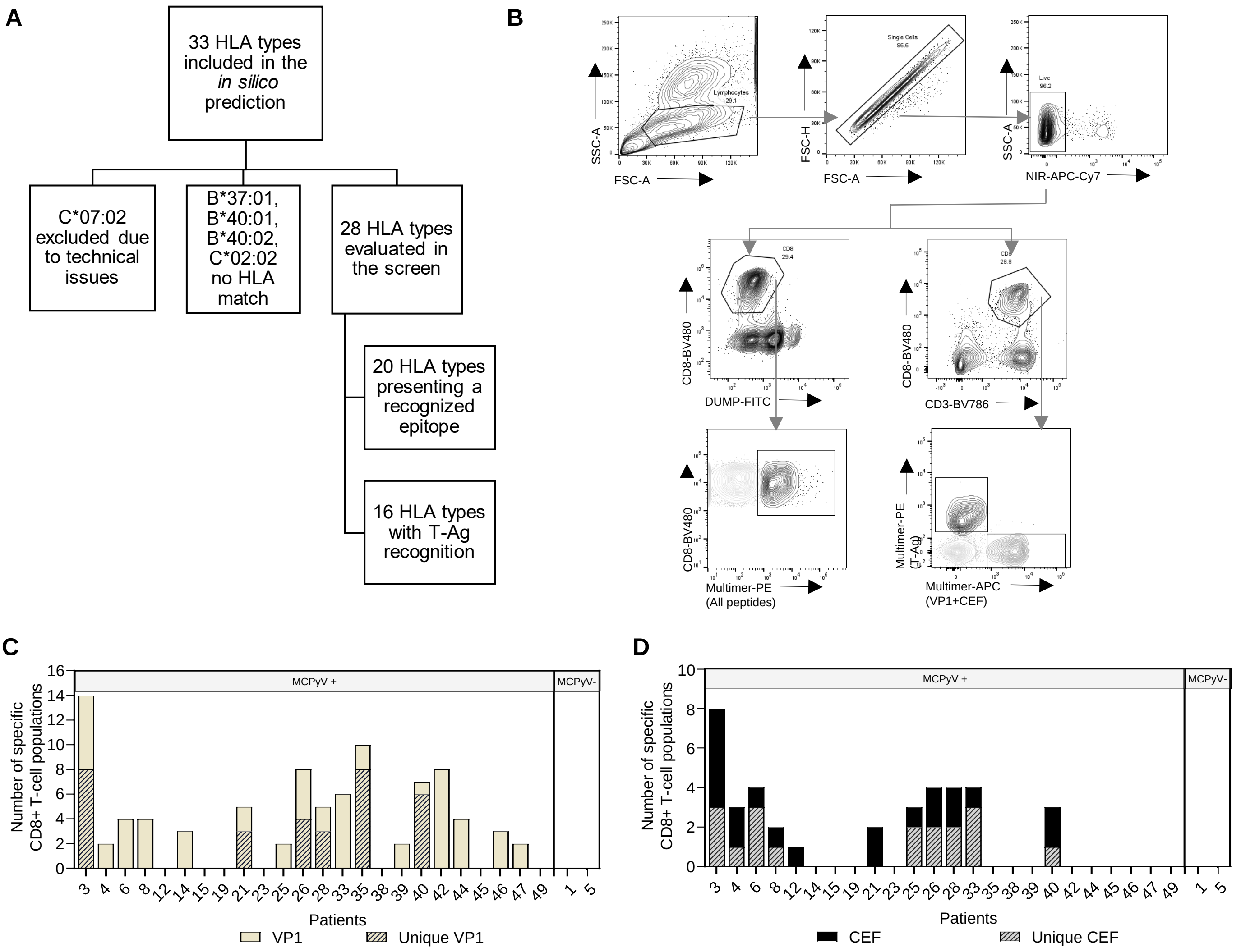

Figure S1. Additional screening results

(A) Flow chart of the HLA haplotypes included in the screening. **(B)** Full gating strategy for sorting of DNA-barcoded multimer-positive T cells. **(C)** The number of VP1-specific T-cell populations detected for the patients with the shaded area indicating the unique epitopes. **(D)** The number of CEF-specific T-cell populations detected for the patients with the shaded area indicating the unique epitopes.

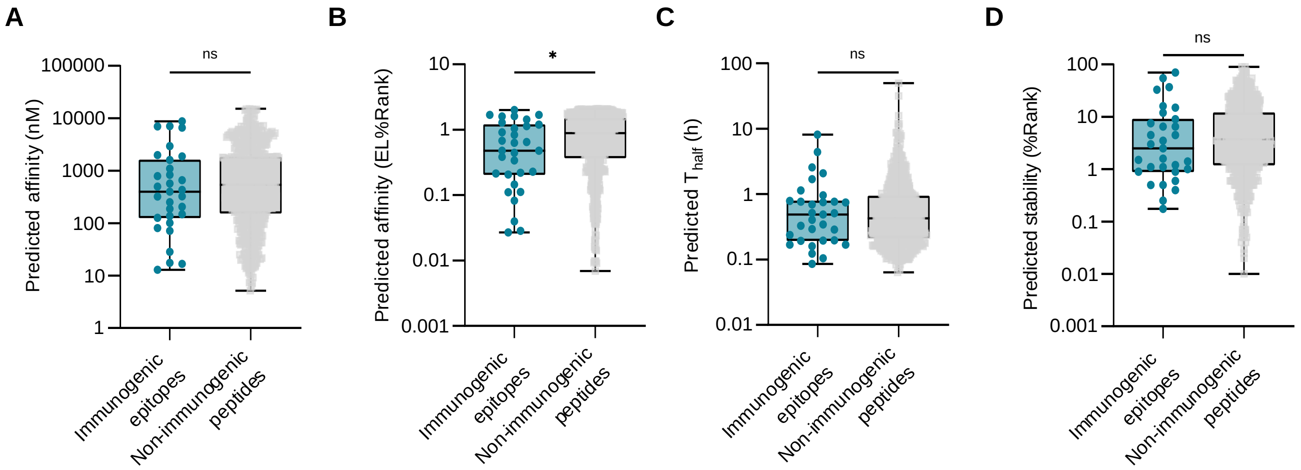

Figure S2. MHC binding predictions of T-Ag peptides

(A-B) Comparison of immunogenic (n=32) and non-immunogenic (n=598) T-Ag peptides in terms of predicted MHC binding affinity using netMHCpan 4.0 given as either predicted affinity **(A)** or eluted ligand percentile rank **(B)**. Mann-Whitney U-test, *p < 0.05. **(C-D)** Comparison of immunogenic (n=32) and non-immunogenic (n=598) T-Ag peptides in terms of predicted MHC binding stability using netMHCstabpan 1.0 given as either predicted half-life of the pMHC complex (T^{half}) **(C)** or percentile rank score of predicted stability **(D)**. Mann-Whitney U-test. All graphs are presented with box plots displaying the interquartile range.

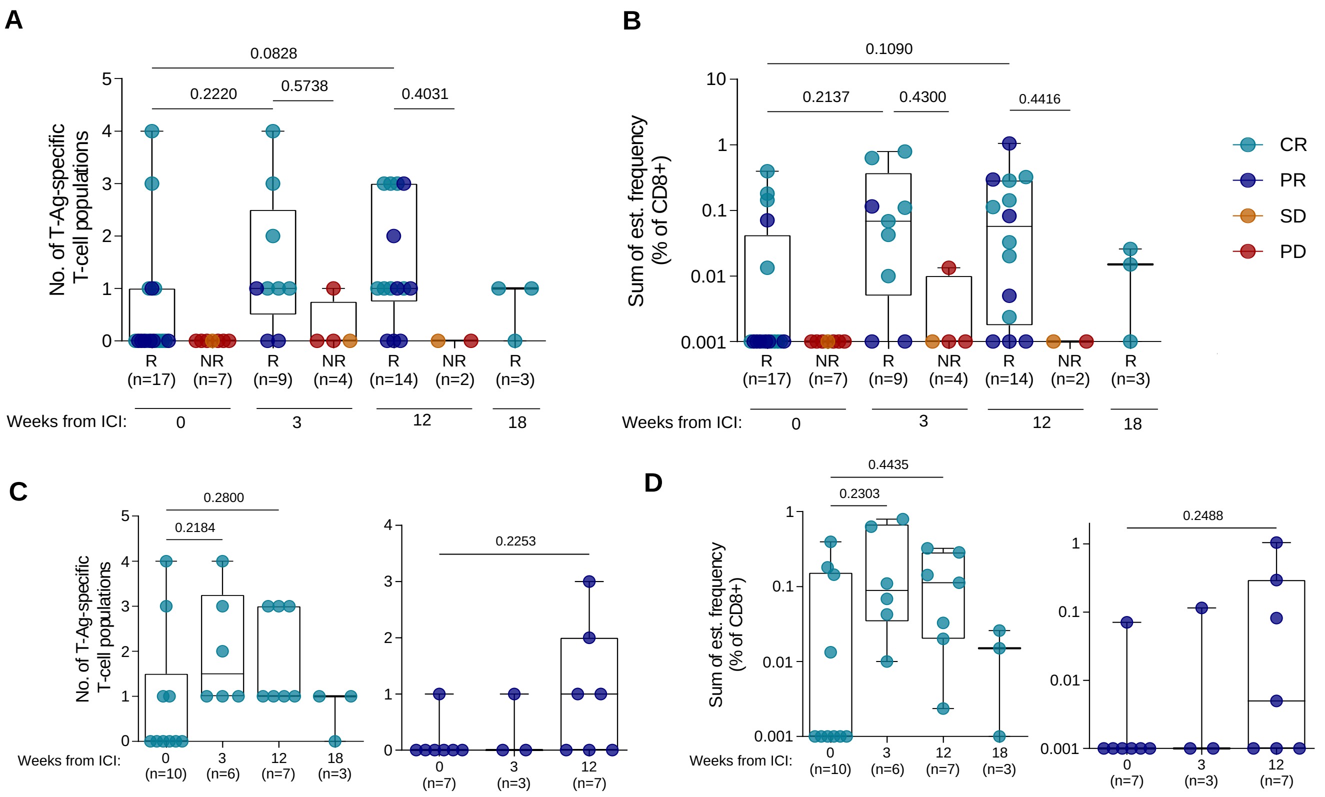

Figure S3. T-Ag-reactive T cells at all individual time points

(A) The number of T-Ag-reactive T cells detected at each time point during ICI therapy for responders (R; CR and PR) and non-responders (NR; SD and PD). Kruskal-Wallis test with Dunn's correction, significance levels annotated. **(B)** The sum of estimated (est.) frequency of T-Ag-reactive T cells at all time points for responders (R) and non-responders (NR). Kruskal-Wallis test with Dunn's correction. **(C-D)** The responder group split into CR patients (left) and PR patients (right) presenting either the number of T-Ag-specific populations **(C)** or the sum of est. frequency **(D)** at each time point. Kruskal-Wallis test with Dunn's correction, significance levels annotated. All graphs are presented with box plots displaying the interquartile range.

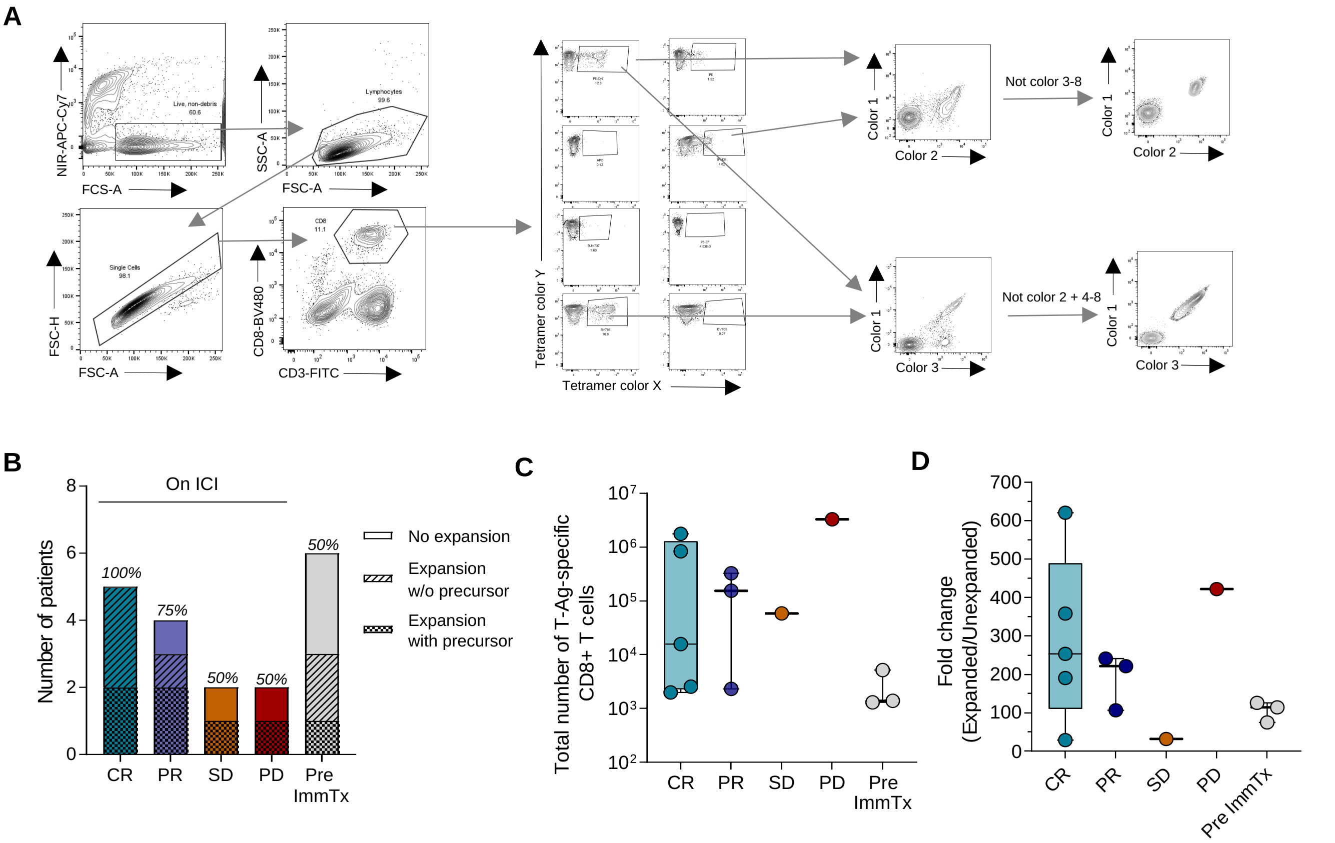

Figure S4. Additional scaffold expansion results

(A) The gating strategy for revealing pMHC specificities of expanded and unexpanded cells. **(B-D)** The 19 patients included in the in vitro Ag-scaffold expansion were divided according to RECIST as complete responder (CR, n=5), partial responder (PR, n=4), stable disease (SD, n=2), or progressive disease (PD, n=2), and a group with blood drawn prior to any immunotherapy (Pre ImmTX, n=6). **(B)** The number of patients' samples expanded from each group with successful T-Ag expansion with or without (w/o) precursors highlighted. The percentage of successful expansion is given above the bars. **(C)** The total number of T-Ag-specific cells expanded in each patient group. Kruskal-Wallis test with Dunn's correction. Box plots displaying the interquartile range. **(D)** Fold change in the number of T-Ag-specific cells expanded in each patient group. Kruskal-Wallis test with Dunn's correction. Box plots displaying the interquartile range.

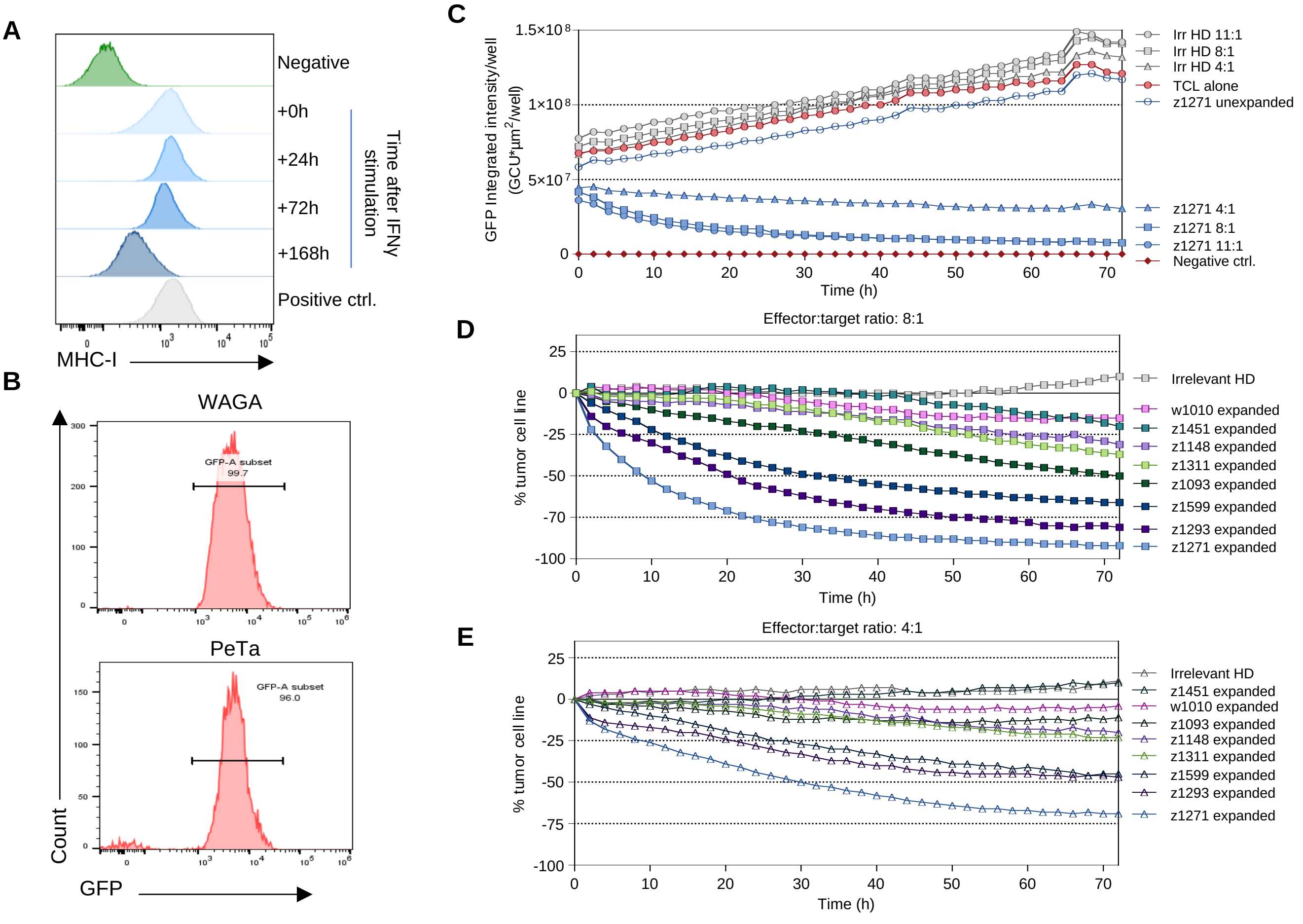

Figure S5. Functional testing

(A) MHC class I levels on WAGA without (negative) or with a 24-hour IFNγ stimulation. After IFNγ removal, the stimulated cells were left incubated for up to 168 hours with MHC class I levels measured after 0h, 24h, 72h, and 168h. Similar results were obtained for PeTa, data not shown. Healthy donor PBMCs were used as a positive staining control. **(B)** GFP levels on transduced WAGA and PeTa. **(C)** Representative curves of the changes in GFP Integrated intensity per well measured in co-culture between tumor cells and unexpanded/expanded cells from patient z1271 (effector:target ratios of 4:1, 8:1, and 11:1), an irrelevant healthy donor (Irr HD), tumor cell line either alone (TCL alone) or in the presence of 1% Triton X-100 (negative ctrl). **(D-E)** Tumor cell line changes during the 72-hour co-culture between HLA-matched tumor cells and expanded patient samples or irrelevant healthy donor (HD) at effector:target ratios of 8:1 **(D)** and 4:1 **(E)**.

Patient ID	Tumor viral status	Clinical response to ICI therapy[A]	HLA-A		HLA-B		HLA-C		# of samples included in screen	Blood collection[C]				PFS (months)	OS (months)
3	Postive	CR	A*01:01:01	A*24:02:01	B*08:01:01	B*44:02:01	C*05:01:01	C*07:01:01	3	C01	N/A	C05	C08	54.67	56.76
4	Postive	CR	A*11:01:01	A*26:01:01	B*27:05:02		C*01:02:01	C*06:02:01	4	C01	C02 (*23 days)	C05	C08	34.50	56.63
6	Postive	CR	A*02:01:01	A*03:01:01	B*07:02:01	B*44:02:01	C*05:01:01	C*07:02:01[B]	3	C01	C02 (*31 days)	C05	N/A	50.04	55.84
8	Postive	PR	A*03:01:01		B*07:02:01		C*07:02:01[B]		2	C01	N/A	C05	N/A	8.64	8.64
12	Postive	PR	A*03:01:01	A*31:01:02		B*44:03:01	C*04:01:01		2	C01	N/A	C05	N/A	23.72	53.18
14	Postive	CR	A*11:01:01	A*68:01:02	B*15:01:01	B*44:02:01	C*01:02:01		1	C01	N/A	N/A	N/A	20.86	34.74
15	Postive	PD	A*01:01:01	A*03:01:01	B*08:01:01		C*07:01:01		1	C01	N/A	N/A	N/A	2.20	3.19
19	Postive	PD	A*02:01:01		B*07:02:01	B*15:01:01	C*07:02:01[B]		1	C01	N/A	N/A	N/A	1.22	2.04
21	Postive	CR	A*01:01:01	A*24:02:01	B*07:02:01		C*04:01:01	C*07:02:01[B]	2	C01	N/A	C05	N/A	25.10	50.19
23	Postive	PD	A*01:01:01	A*03:01:01	B*51:01:01		C*01:02:01		1	C01	N/A	N/A	N/A	1.41	8.64
25	Postive	CR	A*01:01:01	A*03:01:01	B*51:01:01	B*13:02:01	C*06:02:01		2	C01	N/A	N/A	C08	47.87	47.89
26	Postive	PR	A*01:01:01	A*68:02:01		B*57:01:01	C*04:01:01	C*06:02:01	2	C01	N/A	C05	N/A	38.41	46.24
28	Postive	PR	A*01:01:01	A*32:01:01	B*08:01:01		C*07:01:01		2	C01	N/A	C05	N/A	16.82	41.81
33	Postive	PD	A*02:01:01		B*07:02:01	B*15:01:01	C*07:02:01[B]		2	C01	C02	N/A	N/A	2.76	7.99
34	Negative	CR	A*01:01:01						3	C01	C02	C05	N/A	9.00	27.51
35	Postive	CR		A*30:01:01	B*13:02:01	B*51:01:01	C*06:02:01		3	C01	C02	C05	N/A	35.38	35.46
38	Postive	PD	A*24:02:01	A*30:02:01	B*44:02:01		C*01:02:01	C*07:01:01	3	C01	C02	C05	N/A	2.83	26.82
39	Postive	PD		A*32:01:01	B*44:02:01		C*05:01:01	C*06:02:01	2	C01	C02	N/A	N/A	1.64	19.33
40	Postive	SD	A*01:01:01	A*03:01:01	B*35:01:01	B*57:01:01	C*04:01:01	C*06:02:01	3	C01	C02	C05	N/A	5.95	16.63
42	Postive	PR	A*03:01:01	A*68:01:02	B*51:01:01				3	C01	C02	C05	N/A	19.35	31.75
44	Postive	CR	A*03:01:01	A*11:01:01	B*07:02:01		C*07:02:01[B]		3	C01	C02	C05	N/A	23.36	31.62
45	Postive	CR	A*24:02:01	A*68:01:01			C*04:01:01		3	C01	C02	C05	N/A	14.06	30.86
46	Postive	CR	A*24:02:01	A*31:01:02	B*07:02:01	B*35:01:01	C*01:02:01	C*07:02:01[B]	2	C01	C02	N/A	N/A	29.54	30.89
47	Postive	PR	A*11:01:01	A*24:02:01		B*18:01:01	C*03:04:01	C*07:01:01	3	C01	C02	C05	N/A	9.66	9.66
49	Postive	PR	A*01:01:01				C*04:01:01		3	C01	C02	C05	N/A	30.19	30.20
50	Negative	PR	A*26:01:01				C*07:01:01		3	C01	C02	C05	N/A	29.47	29.48

Supplementary Table S2: Cohort 1 patient information with HLA haplotypes included and available peripheral blood samples**.** [A]RECIST criteria obtained following ICI therapy as complete response (CR), partial response (PR), stable disease (SD) and progressive disease (PD). [B]The HLA haplotype C:07:02 was excluded due to technical issues. [C]Blood collection in relation to start of immune checkpoint inhibition (ICI) therapy; C01: pre; C02: 3 weeks; C05: 12 weeks; C08: 18 weeks; N/A: samples not available. PFS: progression-free survival; OS: overall survival.

Patient ID	Tumor viral status	HLA-A		HLA-B		ICI therapy[A]	Days from ICI initiation to blood collection	Clinical response to ICI therapy[B]
w1002	Positive	A*01:01	A*03:01	B*07:02	B*08:01	Avelumab	53	CR
w1010	Positive	A*01:01	A*03:01	B*08:01		N/A	-	N/A
z1082	Positive	A*01:01	A*02:01	B*08:01		N/A	-	N/A
Z1093	Positive	A*02:01				Nivolumab	88	CR
Z1148	Positive	A*03:01		B*08:01		Pembrolizumab	63	CR
z1253	Positive	A*03:01				Pembrolizumab	190	PR
Z1271	Positive	A*02:01	A*24:02			Pembrolizumab	20	PD
Z1293	Positive	A*02:01	A*24:02			Pembrolizumab	66	CR
z1311	Positive	A*01:01				N/A	-	N/A
z1320	Positive	A*01:01				Avelumab	8	PR
z1368	Positive	A*01:01	A*02:01			Avelumab	35	PD
z1369	Positive	A*01:01	A*02:01	B*08:01		Avelumab	98	PR
Z1411	Positive	A*03:01		B*08:01		Pembrolizumab	21	SD
z1428	Positive	A*01:01				N/A	-	N/A
z1440	Positive	A*02:01	A*24:02	B*07:02		N/A	-	N/A
Z1451	Positive	A*02:01				Pembrolizumab	77	CR
z1472	Positive	A*02:01				N/A	-	N/A
z1513	Positive	A*24:02		B*07:02		Pembrolizumab	22	SD
Z1599	Positive	A*01:01	A*02:01	B*08:01		Pembrolizumab	12	PR

Supplementary Table S3: Cohort 2 patient information with HLA haplotypes included and available peripheral blood samples. [A]Immune checkpoint inhibition therapy received; N/A: samples prior to ICI initiation. [B]RECIST criteria obtained following ICI therapy as complete response (CR), partial response (PR), stable disease (SD) and progressive disease (PD); N/A: samples prior to ICI initiation.

Panel	Reagent	Source	Catalog #	Staining type
Antibody panel I:	BV480 anti-human CD8	BD Bioscience	566121.00	Surface
	FITC anti-human CD4	BD Bioscience	345768.00	Surface
	FITC anti-human CD14	BD Bioscience	345784.00	Surface
	FITC anti-human CD19	BD Bioscience	345776.00	Surface
	FITC anti-human CD40	Serotech	MCA1590F	Surface
	FITC anti-human CD16	BD Bioscience	335035.00	Surface
Antibody panel II:	BV480 anti-human CD8	BD Bioscience	566121.00	Surface
	BV786 anti-human CD3	BD Bioscience	563799.00	Surface
	BV650 anti-human CD4	BD Bioscience	563876.00	Surface
	BV711 anti-human CD45RA	BD Bioscience	563733.00	Surface
	FITC anti-human CCR7	Biolegend	353215	Surface
	PE-CF594 anti-human CD39	BD Bioscience	563678	Surface
	BV421 anti-human PD-1	Biolegend	329920	Surface
	BUV737 anti-human CD71	BD Bioscience	749295	Surface
	BV605 anti-human CD28	BD Bioscience	562976	Surface
	APC-R700 anti-human HLA-DR	BD Bioscience	565127	Surface
	BB700 anti-human TCF1	BD Bioscience	564217	Intranucleus
	BUV395 anti-human Ki67	BD Bioscience	564217	Intranucleus
Streptavidin (SA)-conjugated fluorochromes:	SA-PE	Biolegend	405204	Surface
	SA-APC	Biolegend	405207	Surface
	SA-BUV737	BD Bioscience	612775	Surface
	SA-BV786	BD Bioscience	563858	Surface
	SA-PE-Cy7	BD Bioscience	557598	Surface
	SA-BV421	BD Bioscience	563259	Surface
	SA-PE-CF594	BD Bioscience	562284	Surface
	SA-BV605	BD Bioscience	405229	Surface
	SA-BV480	BD Bioscience	564876.00	Surface
Antibody panel III:	BV480 anti-human CD8	BD Bioscience	566121.00	Surface
	FITC anti-human CD3	BD Bioscience	561807	Surface
Antibody panel IV:	PerCP-Cy5.5 anti-human CD8	BD Bioscience	560662	Surface
	BV786 anti-human CD3	BD Bioscience	563799	Surface
	BV711 anti-human CD45RA	BD Bioscience	563733	Surface
	FITC anti-human CCR7	Biolegend	353215	Surface
	PE-CF594 anti-human CD39	BD Bioscience	563678	Surface
	BV421 anti-human PD-1	Biolegend	367422	Surface
	BV650 anti-human CD28	BD Bioscience	740593	Surface
	BV605 anti-human CD27	Biolegend	302830	Surface
	PE-Cy7 anti-human CD57	Biolegend	393310	Surface
	PE anti-human TCF1	BD Bioscience	564217	Intranucleus
	BUV395 anti-human Ki67	BD Bioscience	564217	Intranucleus
	APC anti-human TOX	Miltenyi Biotec	130-118-335	Intranucleus
	AF700 anti-human GZMb	Biolegend	372222.00	Intranucleus
Antibody panel V:	FITC anti-human CD3		561807	Surface
	PerCP-Cy5.5 anti-human CD8	BD Bioscience	560662	Surface
	PE-Cy7 anti-human TNFα	Biolegend	502930	Intracellular
	APC anti-human IFNγ	BD Bioscience	554702	Intracellular

Supplementary Table S4: Panels of reagents used in flow cytometry.
